# Unlocking the hidden chemical space in cubic-phase garnet solid electrolyte for efficient quasi-all-solid-state lithium batteries

**DOI:** 10.1038/s41467-022-35287-1

**Published:** 2022-12-10

**Authors:** Sung-Kyun Jung, Hyeokjo Gwon, Hyungsub Kim, Gabin Yoon, Dongki Shin, Jihyun Hong, Changhoon Jung, Ju-Sik Kim

**Affiliations:** 1 Battery Material Lab, Material Research Center, Samsung Advanced Institute of Technology (SAIT), Samsung Electronics Co., Ltd., 130 Samsung-ro, Yeongtong-gu, Suwon-si, Gyeonggi-do 16678 Republic of Korea; 2 School of Energy and Chemical Engineering, Ulsan National Institute of Science and Technology (UNIST), 50 UNIST‐gil, Ulsan, 44919 Republic of Korea; 3 Neutron Science Center, Korea Atomic Energy Research Institute (KAERI), 111 Daedeok‐daero 989 Beon‐Gil, Yuseong‐gu, Daejeon 34057 Republic of Korea; 4 Energy Materials Research Center, Korea Institute of Science and Technology (KIST), Seoul, 02792 Republic of Korea; 5 Analytical Engineering Group, Material Research Center, Samsung Advanced Institute of Technology (SAIT), Samsung Electronics Co., Ltd., 130 Samsung-ro, Yeongtong-gu, Suwon-si, Gyeonggi-do 16678 Republic of Korea

**Keywords:** Batteries, Characterization and analytical techniques, Materials for energy and catalysis, Solid-state chemistry, Energy

## Abstract

Garnet-type Li_7_La_3_Zr_2_O_12_ (LLZO) solid electrolytes (SE) demonstrates appealing ionic conductivity properties for all-solid-state lithium metal battery applications. However, LLZO (electro)chemical stability in contact with the lithium metal electrode is not satisfactory for developing practical batteries. To circumvent this issue, we report the preparation of various doped cubic-phase LLZO SEs without vacancy formation (i.e., Li = 7.0 such as Li_7_La_3_Zr_0.5_Hf_0.5_Sc_0.5_Nb_0.5_O_12_ and Li_7_La_3_Zr_0.4_Hf_0.4_Sn_0.4_Sc_0.4_Ta_0.4_O_12_). The entropy-driven synthetic approach allows access to hidden chemical space in cubic-phase garnet and enables lower solid-state synthesis temperature as the cubic-phase nucleation decreases from 750 to 400 °C. We demonstrate that the SEs with Li = 7.0 show better reduction stability against lithium metal compared to SE with low lithium contents and identical atomic species (i.e., Li = 6.6 such as Li_6.6_La_3_Zr_0.4_Hf_0.4_Sn_0.4_Sc_0.2_Ta_0.6_O_12_). Moreover, when a Li_7_La_3_Zr_0.4_Hf_0.4_Sn_0.4_Sc_0.4_Ta_0.4_O_12_ pellet is tested at 60 °C in coin cell configuration with a Li metal negative electrode, a LiNi_1/3_Co_1/3_Mn_1/3_O_2_-based positive electrode and an ionic liquid-based electrolyte at the cathode|SE interface, discharge capacity retention of about 92% is delivered after 700 cycles at 0.8 mA/cm^2^ and 60 °C.

## Abstract (web summary)

Conventional compositions of garnet solid electrolytes have limited access to the cubic phase for a high Li content of 7.0, which is beneficial for stability against Li metals. Here, the authors unlock the hidden chemical space via a high entropy strategy, enabling stable long-term battery cycling.

## Introduction

As a promising next-generation battery system, batteries with inorganic solid electrolyte garner considerable attention because it ensures both safety and high energy density owing to the incombustibility of solid electrolyte and the potential feasibility of using metallic lithium as an anode^1^. Specifically, the development of a solid electrolyte with high ionic conductivity and lithium metal stability has been regarded as the gist for realizing the solid-state battery as an alternative to conventional lithium-ion batteries using non-aqueous liquid electrolyte solutions. Among the inorganic solid electrolytes, garnet-type solid electrolyte such as Li_7_La_3_Zr_2_O_12_ (LLZO) is a promising candidate because of its thermodynamic reduction stability against lithium metal as well as its high ionic conductivity of ~1 × 10^−3^ S/cm at 25 °C^2–5^. However, to achieve high ionic conductivity for the garnet-type electrolyte, the stabilization of cubic phase is required between two polymorphs of the cubic (~10^–4^ to ~10^–3^ S/cm at 25 °C) and tetragonal phases (~10^–7^ to ~10^–6^ S/cm 25 °C)^6,7^.

The high ionic conductivity of the cubic phase is caused by the asynchronous motion of single lithium-ion inducing collective motion that requires lower energetic cost for diffusion in contrast to the tetragonal phase exhibiting synchronous fully collective motion^8^. This distinction in lithium conduction mechanism originates from the degree of lithium ordering and the number of available vacancies for lithium diffusion. In particular, the tetragonal phase lacks lithium vacancies and well-ordered lithium site representing Li1 (8a), Li2 (16f), and Li3 (32g) compared to the cubic phase with Li1 (24d) and Li2 (96h) sites with vacancies^9^. However, the adjacent lithium position of Li2 (96h) in cubic phase induces lithium–lithium repulsion that renders the cubic phase energetically unfavorable than the tetragonal phase in case the concentration of lithium increases to seven^10^. Moreover, the tetragonal phase can simultaneously relieve the internal strain energy through the lattice distortion, including the recovery of ZrO_6_ octahedron to a preferred high-symmetry shape and lithium ordering^10^. Although the strain-induced instability of the cubic phase can be resolved at a high temperature, the phase transition from cubic to tetragonal is observed at ~600 °C even in the cooling process after solid-state reaction^11^, which implies that the tetragonal phase is thermodynamically more stable than the cubic phase at ~25 °C if the lithium contents are sufficiently high to induce a nonnegligible repulsion.

Therefore, the cubic phase has been stabilized by forming Li vacancies through aliovalent substitution with dopants such as Al/Ga/Fe for Li sites or Nb/Ta for Zr sites^5,12–16^. Thermodynamically, aliovalent doping is effective for stabilizing the cubic phase by decreasing the Li–Li repulsion as well as increasing the configurational entropy of Li/vacancy ordering, which eventually reduces the Gibbs free energy^10^. The effect of lithium contents on phase stability has been experimentally investigated on Ta-doped LLZO (Li_7–y_La_3_Zr_2–y_Ta_y_O_12_)^14^. For the stabilization of the cubic phase, the experimentally observed critical Li vacancy concentration is almost equal to the theoretically predicted value (*n*_c,vac_ = 0.4–0.5). Below the critical vacancy concentration, the coexistence of cubic and tetragonal phase has been observed in Raman spectroscopy, which exemplifies that the competition between the cubic and tetragonal phases is critically affected by Li and vacancy concentration. As alternative approaches for stabilizing the cubic phase, CO_2_ doping^17^ and high-temperature sintering with alumina crucible had been reported^7^. However, these strategies caused lithium-vacancy formation owing to Li_2_CO_3_ formation or Li evaporation, and unintentional Al doping in Li sites, respectively.

The vacancy formation in garnet-type solid electrolytes induced by the dopant species for stabilizing the cubic phase could be vulnerable to the reduction stability against the lithium metal in terms of lithium chemical potential. As the oxidation or reduction of the solid electrolyte is determined by a kinetically favorable indirect decomposition route via (de)lithiated states instead of the direct pathway forming the thermodynamically equilibrium phase^18^, the vacancy-driven cubic phase with relatively low lithium contents could induce metal reduction suspected as a result of additional lithium accommodation into the crystal structure. Currently, the metal reduction involves undesirable phase transformation of cubic to tetragonal phase or formation of an interfacial layer termed as oxygen-deficient interphase (ODI) layer, which supposedly promotes the increase in interfacial impedance^15,19,20^. Although the lithium accommodation into the crystal structure of garnet has not been directly proven in terms of structure evolution, the formation of tetragonal-like garnet phase is believed as a reduction product by lithiation into the cubic-phase garnet that increases the Li contents in the structure close to 7.0 Li per formula unit. Specifically, the phase transition from the cubic to tetragonal phase upon contact with the lithium metal has been observed under transmission electron microscopy or Raman spectroscopy measurements and analyses^15,19^. The formation of a few nanometers-thick (6–9 nm) tetragonal-like interfacial layer with lithium contents ~7 (from 6.25 to ~6.95) was observed after the contact with the lithium metal through electron energy loss spectroscopy measurements and analyses^19^. Moreover, Fe-doped LLZO displays poorer reduction stability compared to Al-doped LLZO that exhibits a much thicker layer formation of the tetragonal phase (130 μm) and produces a high interfacial resistance (~1 kΩ cm^2^)^15^. Although the dopant (e.g., Fe, Al, Ta, Nb, W, etc.) dependence on interfacial resistance and reduction products (structure and chemical composition) such as tetragonal-like phase or ODI layer needs to be studied in-depth further, it is presumably affected by thermodynamics of intermediate phase and kinetics of the process of forming final decomposition product via indirect decomposition pathway.

Therefore, the metal reduction through phase transformation from the cubic to tetragonal phase or the formation of ODI at the Li|garnet interface should be mitigated to obtain and maintain low interfacial resistance for rapid kinetics of the reversible lithium plating and stripping during charge and discharge cycles. In this context, chemical potential of Li (related to Li vacancy) of solid electrolyte could be a factor to regulate the reduction driving force and kinetics given the assumption that the reduction product is formed via lithiation first following indirect decomposition pathway. This assumption implies that solid electrolyte with higher chemical potential of lithium could be more resistive to reduction against Li metal compared to that with lower chemical potential of lithium. In particular, the stabilization of the cubic phase even at Li-rich reducing environment (at Li ≈ 7) can be a viable strategy for the high reduction stability of garnet against lithium metal. However, the conventional vacancy-driven cubic-phase stabilization could be unable to maintain the original structure when the garnet is exposed to the lithium metal, because the thermodynamic driving force of lithium insertion into the Li-deficient garnet can reduce the vacancy concentration (*n*_c,vac_) below the threshold value of *n*_c,vac_ = 0.4–0.5. Furthermore, as the concentration of vacancy increases, the metal reduction can conveniently occur owing to the low chemical potential of lithium. Therefore, a new approach is required to stabilize the cubic garnet phase under high lithium contents without vacancy formation.

In this study, we report the synthesis and characterization of the cubic-phase garnet with Li = 7.0 composition per formula unit by adopting various dopants above four species on the Zr site without any additional lithium vacancy formation. The entropy-driven stabilization of the cubic-phase garnet decreases the cubic-phase formation temperature from 750 to 400 °C in the solid-state reaction, which implies the potential for low-temperature synthesis. Compared to garnets with low lithium contents, garnets with high lithium contents are more chemically stable against lithium metal in terms of metal reduction kinetics, enabling the cycling of a Li||LiNi_1/3_Co_1/3_Mn_1/3_O_2_ coin cell for 700 times at 0.8 mA/cm^2^ and 60 °C with a discharge capacity retention of about 92%.

## Results and discussion

### Synthesis of cubic garnet with high concentration of lithium

**Fig. 1:**
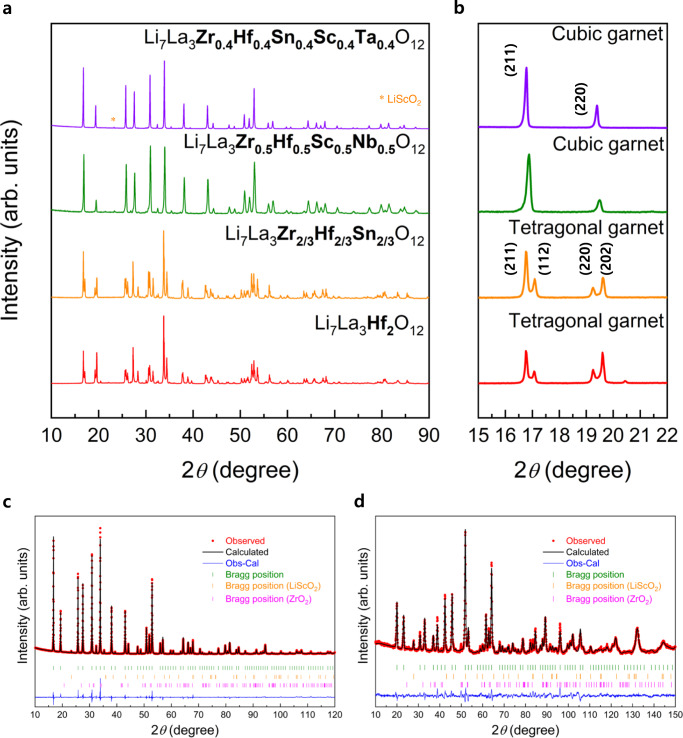
Cubic phase stabilization without vacancy formation. **a**, **b** X-ray diffraction patterns of garnet with respect to number of dopants in Zr site. **c**, **d** X-ray and neutron diffraction patterns and Rietveld refinement of Li_7_La_3_Zr_0.4_Hf_0.4_Sn_0.4_Sc_0.4_Ta_0.4_O_12_.

We synthesized Li_7_La_3_M_2_O_12_ (M = Zr, Hf, Sn, Sc, Ta or Nb) cubic-phase garnet by adopting various dopants in the Zr sites, considering charge balance. In particular, we searched various dopants that can substitute the Zr site given the defect formation energy and preference of dopants in the Zr site (Supplementary Fig. 1)^21^. The site-exchange energy difference in Supplementary Fig. 1 indicates the minimum value of the difference in the defect formation energy of dopants between Zr and Li or Zr and La sites. Therefore, the dopants with low defect formation energy and large difference in site-exchange energy can selectively substitute Zr site. Based on these criteria, the elements in the shaded region (Supplementary Fig. 1) were selected as dopants among the elements with various oxidation states. Specifically, In and Sc were considered as the dopant candidates among the elements with 3^+^ oxidation state. Among the elements with 4^+^ oxidation state, the metal elements Ir, Ru, and Pd were considered including Hf and Sn. Additionally, Nb and Ta, which have been widely applied to the vacancy-driven cubic-phase garnet, were selected because they showed the most favorable to substitute Zr among the elements with 5^+^ oxidation state. Given the average oxidation state obtained with the combinations of dopants, we synthesized the garnet according to the number of dopants in the Zr site. For dopants with 3^+^ oxidation state, we selected Sc instead of In owing to the lower defect formation energy and larger difference in the site-exchange energy. For 4^+^ dopants, Hf and Sn were selected as they are experimentally established for complete substitution of the Zr site (Li_7_La_3_Hf_2_O_12_, Li_7_La_3_Sn_2_O_12_)^22,23^. As displayed in Fig. 1a, pure cubic or tetragonal phases were obtained in case the number of elements in Zr site was above 3 or not exceeding 3, respectively. Both Li_7_La_3_Zr_0.5_Hf_0.5_Sc_0.5_Nb_0.5_O_12_ (C_Zr–Hf–Sc–Nb_) and Li_7_La_3_Zr_0.4_Hf_0.4_Sn_0.4_Sc_0.4_Ta_0.4_O_12_ (C_Zr–Hf–Sn–Sc–Ta_) exhibited the diffraction pattern of cubic phase belonging to Ia$\bar{3}$d space group with lattice parameter *a* = 12.94724(16) Å and 12.92713(63) Å, respectively. Whereas, Li_7_La_3_Hf_2_O_12_ (T_Hf_) as well as Li_7_La_3_Zr_2/3_Hf_2/3_Sn_2/3_O_12_ (T_Zr–Hf–Sn_) were synthesized as tetragonal phase (I4_1_/acd space group) with lattice parameters *a* = 13.12498(11) Å, *c* = 12.63819(13) Å, and the phase was conveniently identified by the diffraction peaks of (211) and (220) around 17° and 19° being separated into (211)/(112) and (220)/(202), respectively, owing to the lower symmetry of this phase compared to the cubic phase (Fig. 1b).

The observations of inductively coupled plasma atomic emission spectroscopy (ICP-AES), Raman spectroscopy, and neutron diffraction (ND) measurements thoroughly proved that the cubic phase can be stabilized at lithium contents ~7 for C_Zr–Hf–Sc–Nb_ and C_Zr–Hf–Sn–Sc–Ta_. Initially, we evaluated and compared the contents of lithium and transition metal in the garnet using ICP-AES by fixing the La contents as 3 per the formula unit of the garnet. The ICP results displayed that all the garnet compounds displayed lithium contents ≥7, regardless of the polymorphs (T_Hf_: 7.32, T_Zr–Hf–Sn_: 7.168, C_Zr–Hf–Sc–Nb_: 7.247, C_Zr–Hf–Sn–Sc–Ta_: 7.609), indicating that the stoichiometric composition of lithium is maintained without evaporation during the synthesis process. The Raman spectrum of C_Zr–Hf–Sn–Sc–Ta_ exhibited no detection of Li_2_CO_3_ that could be typically observed at 1100 cm^–1^, implying that nearly all lithium source participates in forming the garnet phase except little consumption for a few nanometers of Li_2_CO_3_ formation (Supplementary Fig. 2 and Supplementary Notes 1 and 2). Moreover, the relative ratio of amounts of transition metal ion was maintained as target composition (Supplementary Table 1). Furthermore, we precisely confirmed the lithium contents and site occupancy in C_Zr–Hf–Sn–Sc–Ta_ using combined refinement analyses of the X-ray diffraction (XRD) and ND measurements (Fig. 1c, d, and Supplementary Table 2). Overall, the experimentally observed diffraction peaks corresponded appropriately with the calculated model of the cubic-phase garnet with equal occupancy of Zr, Hf, Sn, Sc, and Ta in the Zr site, and almost a pure phase (97.9 %) was obtained with trace amounts of LiScO_2_ (1%) and ZrO_2_ (1.1%) impurities (Supplementary Table 3). Although we considered whether the dopants can occupy the Li site or La site, all dopants selectively substituted the Zr site as expected from the results of first-principle calculation (Supplementary Fig. 1)^21^. The optimized integrated intensity R-factor (*R*_I_ = 4.2%) of ND measurements was achieved at Li ~ 7.0 composition (Supplementary Fig. 3) with nearly half of occupancy in the Li2 (96h) site (0.48) (Supplementary Table 4).

Furthermore, the lithium contents in C_Zr–Hf–Sn–Sc–Ta_ was 7.0 based on the fact that the Li2 (96h) site occupancy was approximately 0.5. Given that the Li2 site is separated into two equivalent positions in the distorted octahedron^9^, the half-occupation of the site indicates the full-occupation in the distorted octahedral site, suggesting that *x* = 6 in Li_x_La_3_M_2_O_12_ denotes the upper limit of Li amount in the octahedral site. In addition, as the amount of lithium in the garnet structure increases, the occupancy of lithium sites with tetrahedral coordination (24d) decreases and those with octahedral coordination (96h) increases^24^. The ND results of C_Zr–Hf–Sn–Sc–Ta_ displayed that 5.76 lithium occupied the Li2 octahedral site in the cubic-phase garnet and the residual 1.24 lithium occupied the Li1 tetrahedral site. This result is in accordance to the trend of preferentially occupying the lithium in the octahedral site as the total amount of lithium per formula unit increases from *x* = 5.0 to *x* = 7.0^25,26^. The high Li2 (96h) site occupancy of C_Zr–Hf–Sn–Sc–Ta_ exceeded that of the vacancy-driven garnet (Li = 6.5, Li2 occupancy = 0.375, corresponding to 4.5 lithium per the formula unit) reported in the literature^24^, indicating that the lithium contents of C_Zr–Hf–Sn–Sc–Ta_ is ~7.

Combined results of ICP, Raman spectroscopy, and Rietveld refinement of XRD and ND measurements indicate that the cubic-phase garnet can be stabilized with the increasing number of dopants in the Zr site without vacancy formation in the Li site. We evaluated the ionic conductivity of the garnet solid electrolyte with Li = 7.0 composition. As expected, the tetragonal phase manifested low ionic conductivity of ~10^–6^ S/cm, whereas C_Zr–Hf–Sc–Nb_ and C_Zr–Hf–Sn–Sc–Ta_ exhibited 2.7 × 10^–4^ and 1.7 × 10^–4^ S/cm bulk conductivity at 25 °C, respectively (Supplementary Fig. 4), which is proximate to the value of Al doped garnet (*σ*_*Li*+_ = 2.11 × 10^–4^ S/cm)^27^.

### Entropy-driven stabilization of cubic phase

**Fig. 2:**
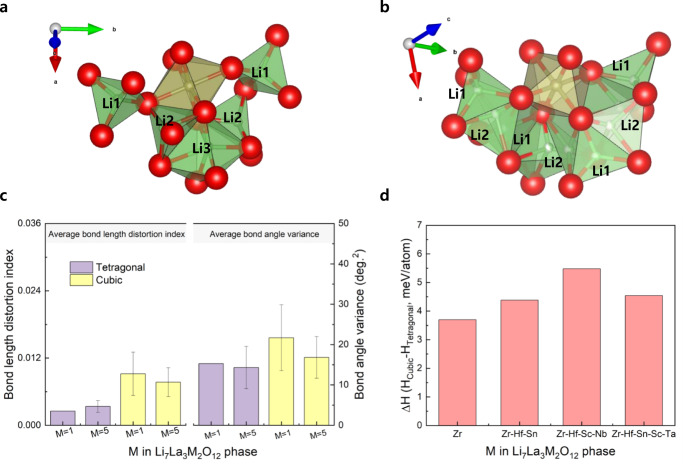
Local structure of Zr site and formation enthalpy with respect to number of dopant species. **a** Local crystal structure of the Zr site with adjacent Li site for the tetragonal phase and **b** the cubic-phase of garnet. Li1 and Li2 sites for the cubic-phase indicate the 24d tetrahedral and 96h octahedral site, respectively. **c** Calculated structural parameters of average bond distortion index and bond angle variance between metal and oxygen in the Zr octahedral site as the tetragonal and cubic phase with Li_7_La_3_M_2_O_12_ composition. **d** Difference in formation enthalpy energy between the cubic and tetragonal phase with respect to number of dopant species.

The stabilization of the cubic-phase garnet presumably originates from the increase in entropy. Previous studies reported that the cubic-phase stabilization in the garnet compound system is an entropy-driven process involving redistribution of lithium ions and lithium vacancies. However, in our system, entropy is predominately increased by the incorporation of multiple distinct metal cations into a crystallographic equivalent site, unlike in the case of the conventional garnet with lithium vacancies^28^. To reveal the origin of the cubic-phase stabilization observed in the multicomponent garnet compound, we first investigated the stability of the cubic and tetragonal phases of the Li = 7.0 composition according to the number of dopants in the Zr site based on the density functional theory (DFT) calculations (Fig. 2). The local strain of the Zr site in two garnet polymorphs, which is an indicator of the degree of Li–Li repulsion varied by the adjacent local environments sharing the corner and edge sites of the octahedral site, was compared according to the number of dopant species (Fig. 2a, b). The structural distortion of the tetragonal and cubic phase was compared based on the variance of both bond length (Zr–O) and bond angle (O–Zr–O) for 16 Zr octahedral site in the supercell to investigate the internal strain impacting the phase stability (Fig. 2c and Table 1)^10^. In particular, the cubic phase experienced more internal strain than the tetragonal phase in terms of both distortion index of bond length and bond angle variance, regardless of the number of dopants as expected. Interestingly, the internal strain of the garnet phase containing five dopant species was relieved in comparison to the cubic phase containing only Zr. In the multicomponent cubic phase, both parameters indicated the internal strain decreased from 0.00918 to 0.00769 for distortion index and from 21.6939° to 16.8122° ^2^. for bond angle variance. However, the tetragonal phases of Zr and Zr–Hf–Sn–Sc–Ta system displayed scarce distinction for the two parameters. These results implied that the internal strain energy required to maintain the cubic phase in the Li = 7.0 composition could be relatively lower for the garnet containing multiple dopant species than the garnet compound with only Zr. Moreover, we compared the enthalpy difference between the tetragonal and cubic phases; in contrast to our expectation, the tetragonal phase was more stable than the cubic phase in all compositions (Fig. 2d). Furthermore, the gap of formation enthalpy between the tetragonal and cubic phase was negligible, regardless of the number of dopants (a few milli electron volts per atom) (Supplementary Table 5). This signified that the incorporation of multiple dopants into the Zr site can mitigate the internal strain, but it is not adequate to stabilize the enthalpy formation energy for the cubic phase of the garnet. Thus, it implies that the cubic phase stabilization in the garnet with multiple dopants is presumably caused by an entropy effect than the enthalpy effect.

**Table 1:** Calculated structural parameters of bond length, distortion index, and bond angle between metal and oxygen in Zr octahedral site at Li = 7.0 composition

Phase	Tetragonal (Zr)	Cubic (Zr)	Tetragonal (Zr–Hf–Sn–Sc–Ta)	Cubic (Zr–Hf–Sn–Sc–Ta)
Bond length (Å)	2.1205	2.1289	2.0992	2.0997
Average Distortion index	0.00254	0.00918	0.00337	0.00769
Average Bond angle variance (deg.^2^)	15.2785	21.6939	14.3168	16.8122

**Fig. 3:**
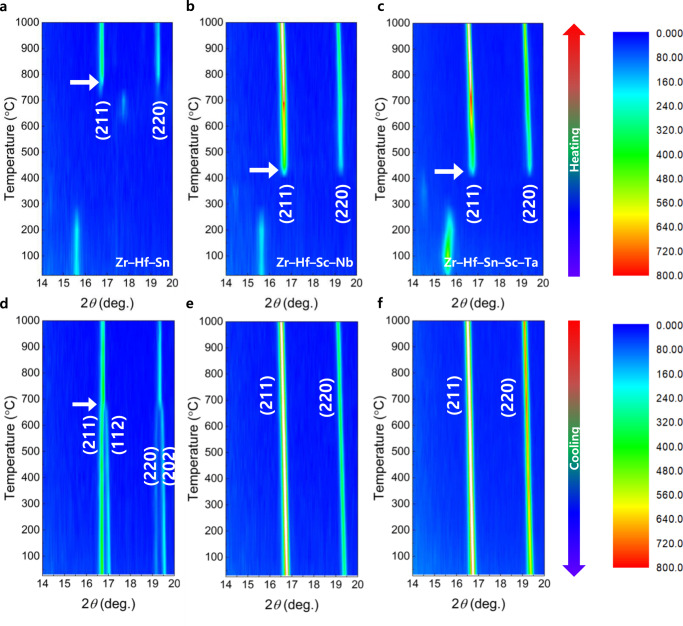
Operando phase evolution during calcination for various numbers of dopants in Zr site. **a–c** Contour plots of X-ray diffraction patterns during heating and **d–f** cooling process in 14–20°. White arrow indicates the temperature of cubic phase formation or phase transition temperature from cubic to tetragonal phase.

As evidence of entropy-driven stabilization, the increase in the number of dopants in the Zr site lowered the nucleation temperature of the cubic phase (Fig. 3 and Supplementary Fig. 5). In addition, we systematically investigated the phase evolution behavior using operando XRD during the calcination process. We acquired XRD patterns during heating and cooling in the temperature range of 25–1000 °C. As depicted in Fig. 3a, the cubic phase formation started over 750 °C for Zr–Hf–Sn system with emergence of the two representative diffraction peaks at 16–17° and 19–20° corresponding to (211) and (220) planes, respectively. The observed cubic formation temperature was analogous to that formed by the solid-state synthetic route^11^. In contrast, the cubic phase formation started from 400 °C for more than four dopants in the Zr site, as portrayed in Fig. 3b, c, and Supplementary Fig. 6. The phase fractions for Zr–Hf–Sn–Sc–Ta system in the temperature range of 450–1000 °C were shown in Supplementary Fig. 7. The lowest temperature was observed in the solid-state reaction forming the cubic phase of garnet, which implied a potential applicability of the garnet with multiple dopants (more than 4) in terms of low-temperature ceramic processing for the production of inorganic solid-state electrolytes for secondary lithium batteries^29^. During the cooling process, we confirmed that the phase transition from the cubic phase to the tetragonal phase was observed at 660 °C for Zr–Hf–Sn system (Fig. 3d), whereas the cubic phase was maintained for the other garnet compounds with four or more dopants in the Zr site (Fig. 3e, f). We expected that Zr–Hf–Sc–Nb or Zr–Hf–Sn–Sc–Ta system would also undergo the cubic–tetragonal phase transition at below the phase formation temperature because of the entropy effect; however, we surprisingly detected that the cubic phase was maintained even at extremely low temperatures until −253 °C (Supplementary Fig. 8). This may be caused by the sluggish kinetics of the cubic–tetragonal phase transition at low temperatures, and in prior research, analogous phenomena have been reported for the entropy-driven stabilized compounds without polymorphic phase transitions^30,31^.

The origin of the entropy-driven stabilization in complex chemical compound such as a garnet system was not apparently caused by the simple increase in the configurational entropy owing to increase in the number of elements in the equivalent crystallographic sites, explained in conventional high-entropy alloy or oxides^32,33^. Because, the change in the configurational entropy of the garnet according to the increase in the number of dopants in the Zr site produces the same amount of increase in both cubic and tetragonal phases. In terms of phonon vibrational entropy, it changes as the number of elements increases in both tetragonal and cubic phase, however, the difference in entropy change between the tetragonal and cubic phase is not relevant, even the entropy increase is slightly larger for the tetragonal phase than the cubic phase (Supplementary Fig. 9). However, the incorporation of various dopants in the Zr site potentially increases the number of accessible microstate basins for the cubic phase at a given energy or a finite temperature. Given that the phonon vibrational and configurational entropy effects were minimal, it can be inferred that the cubic phase stabilization could be due to the increase of other entropy effects such as electronic entropy^34,35^ or electronic configurational entropy effects^36^.

### Ionic conductivity and reduction stability against Li metal

**Fig. 4:**
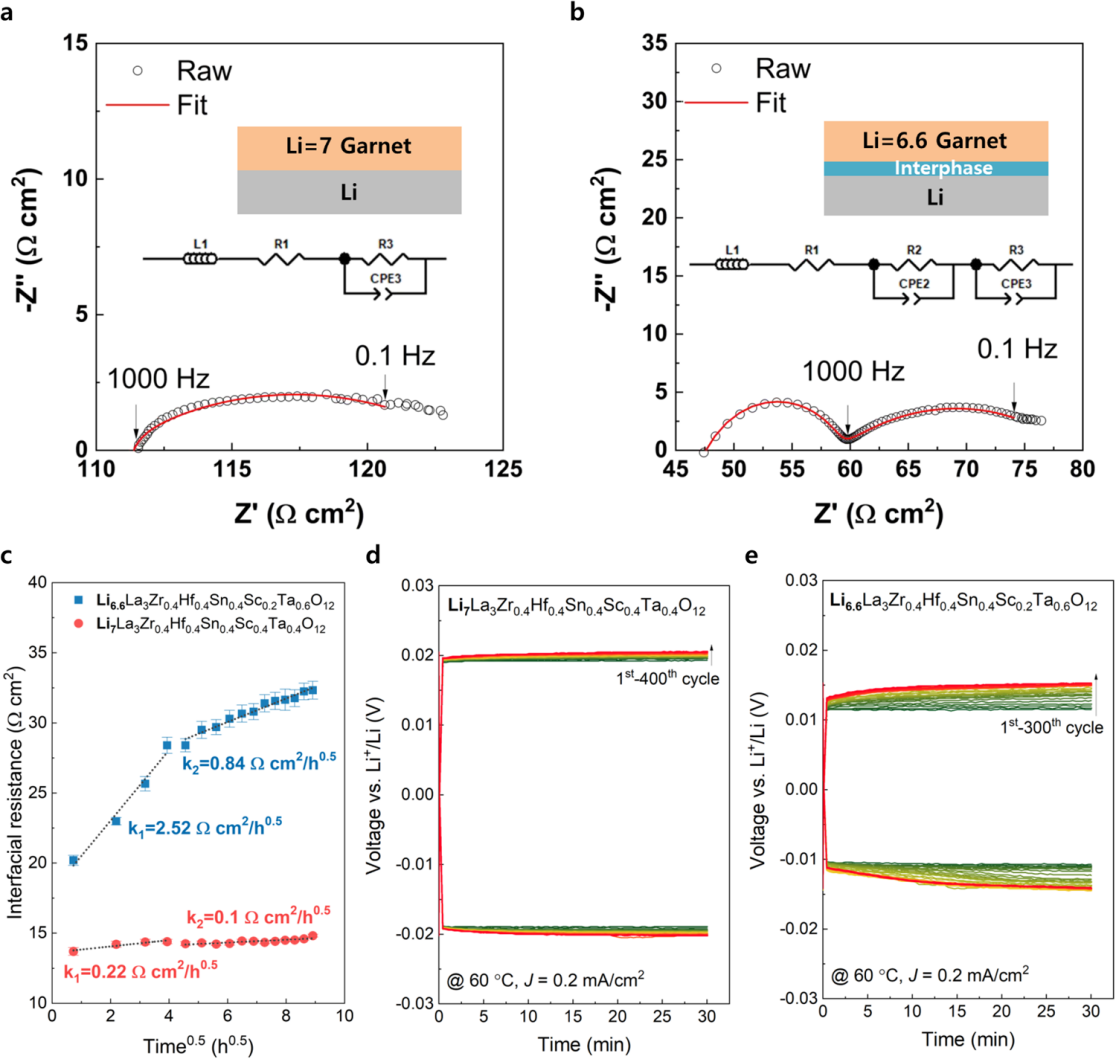
Electrochemical impedance variations in entropy-driven cubic-phase garnet in Li||Li symmetric cell for different lithium contents. **a**, **b** Nyquist plots for the coin-type Li||Li symmetric cells with Li_7_La_3_Zr_0.4_Hf_0.4_Sn_0.4_Sc_0.4_Ta_0.4_O_12_ and Li_6.6_La_3_Zr_0.4_Hf_0.4_Sn_0.4_Sc_0.2_Ta_0.6_O_12_ garnet at 60 °C without additional external pressure. Inset figures describe equivalent circuit model and scheme of interfacial layer structure with Li metal. **c** Variation in interfacial resistance for Li||Li symmetric cells as a function of time at 60 °C (time interval between measurements: 32 min). **d** Li stripping and plating profile of Li_7_La_3_Zr_0.4_Hf_0.4_Sn_0.4_Sc_0.4_Ta_0.4_O_12_ and **e** Li_6.6_La_3_Zr_0.4_Hf_0.4_Sn_0.4_Sc_0.2_Ta_0.6_O_12_ at 60 °C with a current density (*J*) of 0.2 mA cm^–2^.

To evaluate the effect of high lithium contents (Li = 7.0) in the cubic-phase garnet on ionic conductivity and the reduction stability against lithium metal, we compared the ionic conductivity and the reduction stability of two cubic-phase garnets with varying lithium contents (Fig. 4). First, we synthesized a cubic-phase garnet with Li = 6.6 composition (Supplementary Fig. 10) with identical atomic species (Zr/Hf/Sn/Sc/Ta) and increased the relative atomic ratio of Ta/Sc from 1 to 3 (Li_6.6_La_3_Zr_0.4_Hf_0.4_Sn_0.4_Sc_0.2_Ta_0.6_O_12_) for comparison, because the reduction stability is dependent as the type of dopant species^20^. The Rietveld refinement result of ND exhibited the optimized Bragg-R factor (*R*_I_ = 2.41 %) at the target composition (Li~6.6) and confirmed a pure cubic phase (99.0%) with lattice parameter 12.91462(11) Å (Supplementary Figs. 11 and 12, and Supplementary Tables 6 and 7). The relaxation of internal strain, caused by the vacancy formation for relieving the Li–Li repulsion, was confirmed from Williamson–Hall plot (Supplementary Fig. 13).

Interestingly, the bulk ionic conductivity of the Li = 6.6 garnet was confirmed as 3.2 × 10^−4^ S/cm at 25 °C, as displayed in Supplementary Fig. 10, which is 88% higher than that of the Li = 7.0 garnet (1.7 × 10^−4^ S/cm at 25 °C). Several studies have studied the correlation between the amount of lithium and ionic conductivity. Goodenough’s group argued that the formation of vacancy in octahedral sites contributed to high ionic conductivity, and a 3:1 ratio of Li^+^ occupancy/vacancy in those sites would be the optimal value in terms of fast ionic conduction^37^. Sakamoto’s group rather insisted that increasing the lithium amount along with the 96h site occupancy enhances the ionic conductivity. This is because the high occupation of lithium ions in the 96h site can increase the number of effective charge carrier caused by destabilization of the lithium ions on the more mobile 96h site owing to the strong Coulombic repulsion at the reduced 24d–96h distance^24^. However, the correlation between the lithium/vacancy concentration and the ionic conductivity was unclear, especially for the cubic-phase garnet with the high lithium contents (>6.6) because its experimental implementation of the high lithium contents garnet remained a challenge until so far. In the high-entropy cubic-phase garnet system offering a lithium-stuffed environmental with Li = 7.0 composition, the ND results confirmed the highest occupancy of lithium in the octahedral 96h site (96%) among the reports on the cubic-phase garnets. In the Li > 6.6 composition in the cubic-phase garnet, the Li = 7.0 garnet exhibited nearly same 24d–96h distance (1.593(14) Å) with that of the Li = 6.6 garnet (1.58905(0) Å), despite displaying a high 96h site occupancy in comparison to the Li = 6.6 garnet (84%) (Supplementary Tables 4 and 7). This resulted in similar activation energy for both two different garnets (406.8 meV and 403.5 meV for the Li = 7.0 and Li = 6.6 garnet, respectively) (Supplementary Fig. 10b), which implies that in the case of garnet system with high lithium contents (Li > 6.6), the high occupation of lithium ions in the octahedral site has little effect on change of energy landscape for diffusion, rather, the increase of the number of vacancy for lithium interstitial site directly affect the increase in the prefactor of ionic conductivity.

After assembling the symmetric cell (Li|SE|Li) for the two garnet compounds with different lithium contents by applying cold isostatic pressure of 250 MPa, we measured the electrochemical impedance spectroscopy (EIS) variations at 60 °C with respect to time (Fig. 4a–c) to investigate the reduction stability. EIS spectra exhibit a depressed semicircle at low frequencies below 1000 Hz for both compositions of Li = 7.0 (Fig. 4a) and Li = 6.6 (Fig. 4b), and an additional semicircle is observed only in case of the Li = 6.6 garnet at high frequencies over 1000 Hz. The enlarged EIS spectrum of the Li = 7.0 garnet clearly showed that no semicircle appeared at high frequencies region over 1000 Hz (Supplementary Fig. 14). According to the equivalent capacitance value of constant phase element (*Q*_CPE_ = 9.2 × 10^−3^ F s^α−1^) at low frequencies, which is considered as the electrochemical reaction contribution^38,39^, the corresponding resistance is apparently induced by the barrier for Li diffusion including the charge transfer process (R_3_CPE_3_)^20^. The detailed fitting parameters are shown in Supplementary Table 8. The depressed semicircle at high frequencies induced by the interfacial layer (R_2_CPE_2_) is observed only in the Li = 6.6 garnet given the capacitance value of constant phase element (*Q*_CPE_ = 5.6 × 10^−7^ F s^α−1^) and the negligible grain boundary resistance of sintered pellet (Supplementary Fig. 15). Considering the absence of interfacial resistance for the Li = 7.0 garnet and the high isostatic pressure up to 250 MPa for improved Li|SE adhesion, the interfacial resistance shown in the Li = 6.6 garnet was not caused by the constriction resistance regarding the contact geometry^40^ but owing to the metal reduction interphase (MRI) layer formed after the chemical reaction with lithium^20,41^. According to the DFT calculations, the thermodynamic reduction potentials for the Li = 7.0 and Li = 6.6 garnets displayed similar results of 1.34 and 1.45 V, respectively, owing to the reduction of Sn (Supplementary Fig. 16). In contrast, the reaction energy of the garnet with Li metal (at 0 V) was lower in case of the Li = 7.0 garnet (137.4 meV/atom) than the Li = 6.6 garnet (162.9 meV/atom), thereby indicating that the garnet with high lithium contents could exhibit higher kinetic barrier for metal reduction by lithium metal in comparison to the garnet with less lithium contents. Therefore, this finding implies that the kinetic barrier required for forming the MRI layer is dependent on the lithium composition in the garnet system. In particular, the chemically formed MRI layer increases the resistance at low frequencies as a function of time. Moreover, the bulk (*R*_1_) and interfacial resistance (*R*_2_) is almost maintained without significant variations for both Li = 7.0 and Li = 6.6 garnets. However, the diffusional resistance including the charge transfer (*R*_3_) increased from 20 to 32.5 Ω cm^2^ for the Li = 6.6 composition after 110 h, whereas that of the Li = 7.0 composition displayed a slight variation from 13.8 to 14.6 Ω cm^2^. Overall, the rate constant of increase in the resistance per square root of time for the Li = 6.6 garnet (0.84 Ω cm^2^/h^0.5^) was eight times higher than that of the Li = 7.0 garnet (0.1 Ω cm^2^/h^0.5^) after 16 h, thereby implying that the cubic-phase garnet with high lithium contents in the structure is kinetically stable against lithium metal.

During stripping and plating of the lithium metal, the Li = 6.6 garnet exhibited a greater increase in overpotential than the Li = 7.0 garnet, as displayed in Fig. 4d, e. To minimize the resistance variations originating from the morphological transformation or void formation at the Li|SE interface^40,42^, the lithium stripping and plating were performed at high temperature (60 °C) with a low current density of 0.2 mA/cm^2^ for 30 min, corresponding to an areal capacity of 0.1 mAh/cm^2^. The initial overpotential of the Li = 6.6 garnet composition was small in comparison to that of the Li = 7.0 garnet owing to its relatively high ionic conductivity. During repeated 400 cycles of the lithium plating and stripping, the Li = 7.0 garnet displayed only negligible changes in the overpotential of ~ 1 mV, whereas the Li = 6.6 garnet exhibited a sizeable increase in the overpotential of ~4 mV. Although the absolute value of increase in the overpotential of the Li = 6.6 garnet appeared as small, it cannot be neglected, because the overpotential significantly increased by 36% from the initial value. In addition, the impact was even greater, considering the high practical values required for practical applications, such as a large electrode area (~200 cm^2^), thin solid electrolyte (30 μm), high current density (5 mA cm^–2^), and wide operating temperature range (−20–100 °C)^43^.

Furthermore, the ex situ X-ray photoelectron spectroscopy (XPS) measurements and analyses confirmed that the Li = 6.6 garnet was more vulnerable in terms of the reduction stability against lithium metal compared to the Li = 7.0 garnet. We performed XPS analysis on five metal core levels (La 3*d*, Zr 3*d*, Hf 4*f*, Sn 3*d*, and Sc 2*p*) for both Li = 7.0 and Li = 6.6 garnets after contact with the lithium metal for the same period, and the results are displayed in Supplementary Fig. 17. The contact with the lithium metal resulted in more reduction of both Zr and Sn for the Li = 6.6 garnet compared to the Li = 7.0 garnet, and other metal species displayed no significant difference between the two garnets except for Zr and Sn. The reduction of Zr^4+^ to Zr^2+^ was evidently observed for the Li = 6.6 composition with the appearance of shoulder peaks at 181.7 eV. In contrast, only a chemical shift to 182.5 eV induced by a slight reduction was observed for the Li = 7.0 garnet. In Sn 3*d* core level, the lower binding energy of Sn 3*d*_5/2_ peak was observed for the Li = 6.6 garnet compared to the Li = 7.0 garnet (485.9 eV for Li = 7.0 garnet vs. 485.3 eV for Li = 6.6 garnet). These results signified that the reduction stability of the garnet is influenced by the lithium contents as well as the dopant species in the garnet system.

Similar trend is also observed in O-K edge spectra via ex situ soft X-ray absorption spectroscopy (sXAS). As shown in Supplementary Fig. 18, the Li = 7.0 garnet showed nearly identical absorption spectra regardless of the contact with the lithium metal, meanwhile, the Li = 6.6 garnet showed a slight increase in the peak intensity around 533.3 eV and in the range of 538.5–539.7 eV after the lithium metal contact. Although the O-K edge spectrum has complex information owing to the various dopants-driven hybridization orbital (Sc-3*d*, Zr/Sn-4*d*, Hf/Ta/La-5*d*, and O 2*p*), evolution in the Li = 6.6 garnet seems to be owing to the formation of MRI layer induced by Zr and Sn reduction confirmed by XPS. Because there is no clear evidence of the ODI layer formation such as emergence of Li_2_O. In addition, the existence of shoulder peak in both Li = 7.0 and Li = 6.6 garnet (533.3–534.1 eV) even after the contact the with lithium metal corroborates that cubic phase is well maintained without tetragonal phase formation. Absorption peak at low energy around 533.3–534.1 eV for the Li = 6.6 and Li = 7.0 is believed as a feature of cubic phase by comparing the reference spectra of tetragonal-phase garnet (Li_7_La_3_Zr_2/3_Hf_2/3_Sn_2/3_O_12_) and Ta-doped cubic-phase garnet (Li_6.5_La_3_Zr_1.5_Ta_0.5_O_12_). In contrast to the cubic phase, the tetragonal phase showed only absorption peak at 535.2 eV without the peak at lower value, and the absence of shoulder peak (533.3–534.1 eV, shaded region in Supplementary Fig. 18a, b) was also confirmed by EELS analysis^19^. In addition, given the cubic phase was appropriately maintained for both samples as confirmed by the ex situ Raman measurements and analyses (Supplementary Fig. 19), we can infer that the increase of the interfacial resistance was primarily induced by the formation of MRI layer with the metal reduction owing to the contact with the lithium metal without the phase transformation.

### Electrochemical energy storage performance in quasi-all-solid-state cell configuration

The battery performance of the quasi-all-solid-state cells was evaluated using the Li = 7.0 and the Li = 6.6 garnet solid electrolytes. The quasi-all-solid-state coin cell comprises a LiNi_1/3_Co_1/3_Mn_1/3_O_2_-based positive electrode (NCM111) wetter with a non-aqueous ionic liquid-based electrolyte solution (i.e., 2 M Lithium bis(fluorosulfonyl)imide (LiFSI) in *N*-methyl-*N*-propylpyrrolidinium bis(fluorosulfonyl)imide (Pyr_13_FSI)) and the SE in direct contact with a lithium metal negative electrode.

**Fig. 5:**
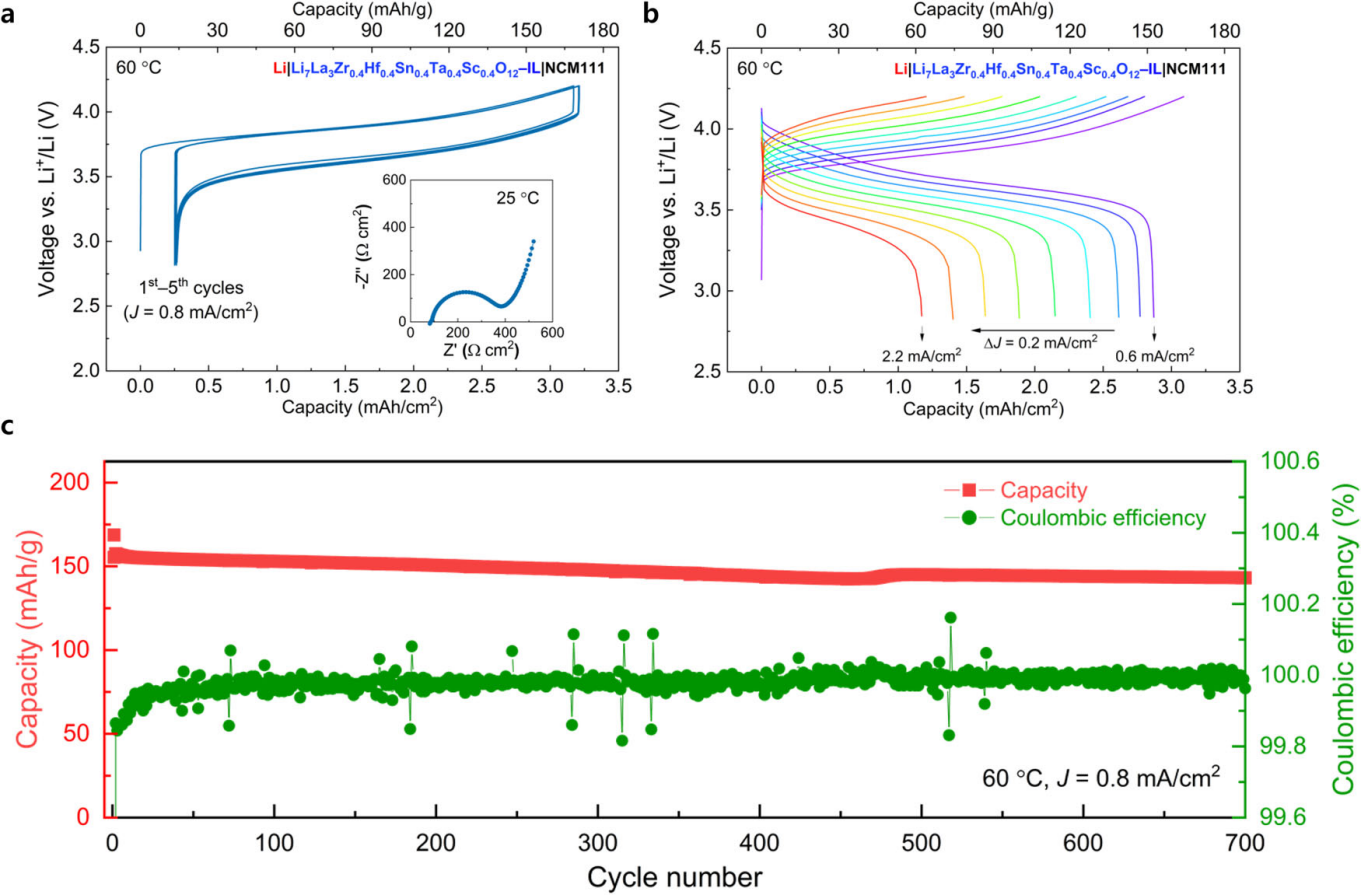
Battery performance of the quasi-all-solid-state Li||NCM111 cells comprising Li_7_La_3_Zr_0.4_Hf_0.4_Sn_0.4_Sc_0.4_Ta_0.4_O_12_ as solid electrolyte. **a** Electrochemical profile of solid-state batteries at 60 °C with a current density (*J*) of 0.8 mA/cm^2^ from first to fifth cycles. Inset is the Nyquist plot of the cell at 25 °C before cycling. **b** Charge/discharge voltage profile with current density (*J*) increasing from 0.6 to 2.2 mA/cm^2^ (∆*J* = 0.2 mA/cm^2^) at 60 °C. **c** Long-term cycling stability performance of the quasi-all-solid-state Li||NCM111 cell at 60 °C and 0.8 mA/cm^2^.

The charge–discharge profiles of the Li metal coin cells containing the Li = 7.0 and Li = 6.6 garnet-type SEs and tested at 60 °C were illustrated in Fig. 5a and Supplementary Fig. 20, respectively. In case of the Li = 6.6 garnet, the initial discharge capacity was 3.0 mAh/cm^2^ at a current density of 0.8 mA/cm^2^ (Supplementary Fig. 20), which was slightly higher than that of the Li = 7.0 garnet (2.92 mAh/cm^2^ in Fig. 5a). This can be explained by the low overpotential originating from the relatively high ionic conductivity of the Li = 6.6 garnet (Supplementary Fig. 10b) compared to that of the Li = 7.0 garnet, as observed during the Li||Li symmetric cell measurements (Fig. 4d, e). In contrast to the result of the symmetric cell test, the short-circuit occurred in the Li = 6.6 garnet after only a few cycles even at a low current density of 0.4 mA/cm^2^ (Supplementary Fig. 20). Thereafter, we repeatedly conducted the cell cycle test for the Li = 6.6 garnet at various current densities. The results revealed the occurrence of the short-circuit in all cells under identical experimental conditions. The experimental discrepancy regarding the short-circuit formation observed in the Li = 6.6 garnet could be explained based on the low current density and the limited amount of lithium participating in the electrochemical reaction used in the Li||Li symmetric cell test. In the symmetric cell, a limited amount of lithium was used at a low current density of 0.2 mA/cm^2^ for 30 min corresponding to a capacity of 0.1 mAh/cm^2^ (Fig. 4e). However, a capacity of ~3.0 mAh/cm^2^ was used with a high current density of 0.8 mA/cm^2^ in the cell evaluation (Supplementary Fig. 20). Therefore, the relatively high current condition and large amounts of repeated lithium plating/stripping in the half-cell experiments could accelerate the dendrite formation at the interface between the electrolyte and electrode. On the other hand, we empirically found that critical current density (CCD) values in the case of Li||Li symmetric cells tended to be underestimated, compared to those of the half-cell experiments. The CCD values are governed by more complicated factors in the Li||Li symmetric cell configuration than in the asymmetric half-cell configuration, because lithium plating/stripping processes causing morphological transformation such as nucleation of lithium metal and its void formation, which influences the increase in interfacial resistance, occur simultaneously on both sides of the solid electrolyte. In addition, the presence of the resistive MRI layer observed only for the Li = 6.6 garnet may lead to early formation of short-circuits. The correlation between MRI layer and the premature short-circuit formation observed in the Li = 6.6 garnet remains a challenge to be elucidated.

The charge–discharge profile of the cell comprising the Li = 7.0 garnet SE was obtained by applying a current density of 0.8 mA/cm^2^ (~42.5 mA/g) within 2.85–4.2 V vs. Li^+^/Li at 60 °C, as illustrated in Fig. 5a. In the first cycle, the cell exhibited an initial discharge capacity of 155 mAh/g corresponding to 2.92 mAh/cm^2^ and 92% Coulombic efficiency. Additionally, we measured the rate performance of the Li = 7.0 garnet by increasing the current density from 0.6 to 2.2 mA/cm^2^ at an interval of 0.2 mA/cm^2^ (Fig. 5b). At a high current density of 2.2 mA/cm^2^, an areal capacity of 1.17 mAh/cm^2^ was obtained, which corresponds to 40% of the capacity value at a low current density of 0.8 mA/cm^2^. After the initial cycling, the cell demonstrated stable cycle performance and maintained a high capacity over 143 mAh/g (~2.69 mAh/cm^2^) until 700 cycles with 92% of capacity retention (Fig. 5c), contrast to that of the Li = 6.6 garnet. The stable cycling performance was a consequence of the reduction stability of the garnet against the lithium metal that kinetically hinders the increase in interfacial resistance, as confirmed from Fig. 4c.

In addition, we applied a surface modification to the Li = 7.0 garnet to maximize its electrochemical behavior and improve the Li||NCM111 cell performances. The surface treatment of the solid electrolyte pellet using acid treatment (1 M HCl for 30 min) was conducted as following reasons: (1) Removal of residual Li_2_CO_3_ layers in a few-nanometer-scale observed from XPS and sXAS measurements (Supplementary Fig. 2). (2) Increase of surface roughness to enlarge the contact area with the lithium metal anode. (3) Surface chemical modification for inhibiting the formation of by-products. The acid-treatment is also known for an effective approach in significantly reducing their interfacial resistance by removing the impurities as well as increasing the active surface area and modifying the composition of the surface^3,44^. No significant changes in ionic conductivity or the ratio between multiple dopants at the Zr site was observed after acid treatment (Supplementary Fig. 21). As depicted in Supplementary Fig. 22, the initial discharge capacity increased to 3.17 mAh/cm^2^ compared to that before the acid-treatment at a current density of 0.8 mA/cm^2^, and a high areal capacity of 2.45 mAh/cm^2^ was obtained at a high rate of 3.0 mA/cm^2^. Moreover, a discharge capacity retention of 86% after 800 cycles at 3.0 mA/cm^2^ and 60 °C was observed (Supplementary Fig. 23). In addition, we demonstrated that the cycle life could be further increased (i.e., discharge capacity retention of 86% after 2000 cycles at 3 mA/cm^2^ and 60 °C) at the expense of the initial reversible capacity to 1.66 mAh/cm^2^ by reducing the exposure time of the acid, as shown in Supplementary Fig. 24. The reversible capacity decreased with a low active surface area between the electrolyte and electrode as the acid treatment time decreased. Thus, the improvement of the cycle life was potentially caused by the reduced irregular surface damage owing to the isotropic characteristic of chemical wet etching, which suggested that precise surface engineering forming a stable interface is vital for improving the electrochemical performance, especially when using a thin solid electrolyte.

In summary, we demonstrated the entropy-driven cubic phase stabilization in garnet-type solid electrolyte (Li_7_La_3_Zr_2_O_12_) by incorporating five dopants in the Zr site. The dopants were selected by comprehensively considering their defect formation energy, site preference, and valence state. Based on XRD, ND, and ICP measurements and analyses, we revealed that the multicomponent high-entropy garnet can stabilize the cubic phase at ambient temperatures at a constant lithium content of 7.0, unlike conventional vacancy-driven cubic-phase garnet compounds with Li ≤ 6.6. Additionally, we detected that the nucleation of the entropy-driven cubic phase initiates at a low temperature of 400 °C, indicating the possibility of low-temperature synthesis. For the garnet system, these high-entropy effects such as the stabilization of the high-temperature phase and reduction of the phase formation temperature were potentially caused by the population of various microstates affecting the electronic or electronic configurational contributions in the total entropy rather than the phonon vibrational and configurational entropy observed in the common high-entropy alloy systems, and thus, further in-depth study is required. Furthermore, the entropy-driven cubic-phase garnet displayed excellent reduction stability against lithium metal compared to the garnet with relatively low lithium contents in the kinetic aspect, and the high lithium amount was maintained without transiting to the tetragonal phase. More importantly, the reduction stability and the increase in interfacial resistance have been relatively overlooked in comparison to the bulk ionic conductivity of solid electrolytes, because the overall resistance is primarily determined by the low bulk ionic conductivity of most solid electrolytes and the relatively large thickness/area ratio in most cell geometries. However, thin solid electrolyte with thickness ≤100 μm should be required considering the energy density, and the proportion of interfacial resistance in the total resistance becomes more significant in such cases. In terms of the lithium chemical potential in the garnet system, we confirmed that the reduction stability was improved by increasing the lithium chemical potential, which formed a stable interface and inhibited the formation of ODI layers. This observation provides clues to the correlation between the formation of ODI layer and dendrite nucleation for future research. Furthermore, the high-entropy garnet can provide a platform to further understand the fundamental parameters governing the lithium ionic conductivity and their correlation in a lithium-stuffed environment by allowing access to a cubic-phase garnet with Li > 6.6 composition, which is currently challenging to experimentally demonstrate owing to the phase instability of the cubic phase. Overall, the current study suggests that the strategy to increase entropy by introducing multiple dopants in a crystallographically equivalent site can be applied to complex systems such as a garnet solid electrolyte, and this aids in further understanding the material properties along with the discovery of novel materials manifesting unexpected characteristics.

## Methods

### Material preparation

Garnet solid electrolytes were synthesized through the solid-state reaction. Accordingly, the precursors of La_2_O_3_ (99.9 %, Thermo Fisher Scientific), ZrO_2_ (99.9%, Thermo Fisher Scientific), HfO_2_ (99.9%, Thermo Fisher Scientific), SnO_2_ (99.99, Sigma Aldrich), Sc_2_O_3_ (99.9%, Thermo Fisher Scientific), Ta_2_O_5_ (99.85%, Thermo Fisher Scientific), and Nb_2_O_5_ (99.9%, Thermo Fisher Scientific) were used in stoichiometric ratios and 10 mol% excess of Li_2_O (99.5%, Thermo Fisher Scientific) was used for synthesis. The precursors were mixed using planetary mill under 300 rpm for 2 h with ball (5 mm zirconia) to powder ratio of 4:1 (Pulverisette-7 Premium Line, Fritsch, Germany). The ball-milling jar was filled with dry air. The resulting mixture of the precursors were calcined in a box furnace at 1000 °C for 12 h (AJ-SB4, Ajeon Furnace Control, Korea) in air atmosphere. After creating a pellet under a uniaxial pressure of 300 MPa, the calcined powder was sintered at 1200 °C for 4 h. Subsequently, a commercially available lithium nickel–cobalt–manganese oxide (NCM111) electrode composed of active material with 12 μm average particle size as secondary particle (96 wt%), conductive carbon (2.2 wt%), and poly(vinylidene fluoride) (PVDF) binder (1.8 wt%) was prepared. Cathode active material powder (NCM111, Samsung SDI), a carbon conductive agent (Super P, 3 M), and a binder (PVDF, Solvay) were uniformly mixed in N-methyl-2-pyrrolidone anhydrous (NMP, Sigma–Aldrich) solvent to prepare slurry. The slurry was casted on an aluminum current collector having a thickness of about 12 μm. Then, the electrode was dried at a temperature of 120 °C for 2 h under vacuum, followed by a rolling process. The active material loading for the positive electrodes was 18.75 mg/cm^2^. In addition, the ionic liquid electrolyte (water content <0.01 wt%) was prepared by dissolving 2 M concentration of LiFSI (99.99%, PANAX ETEC Co., Ltd.) into Pyr_13_FSI (99.9%, Kanto Chemical Co. Inc.) ionic liquid in dry room (dew point, –60 °C). Both LiFSI and Pyr_13_FSI were used as received for preparation of ionic liquid electrolyte. The acid treatment was applied to the garnet surface by simply immersing the sintered electrolyte disc into 1 M HCl solution (in distilled water) in dry room at 25 °C for 5 min. Thereafter, the sintered electrolyte disc was washed with anhydrous ethanol and immediately dried with blowing of dry air.

### Physicochemical characterizations

The composition of the synthesized garnet solid electrolytes was measured by inductively coupled plasma atomic emission spectroscopy (ICP-AES; ICPS-8100, Shimadzu, Japan). The crystal structure of the synthesized garnet solid electrolytes was investigated using X-ray diffraction (XRD), and XRD patterns were obtained using a D8 Discover (Bruker, Germany) diffractometer with Cu–K*α* radiation in the 2*θ* range of 10–90° at 1° min^–1^. For the Rietveld refinement analysis, XRD pattern was acquired using an Empyrean diffractometer (Malvern Panalytical Ltd., UK) with Cu–K*α*1 radiation in the 2*θ* range of 10–120° at 0.5° min^–1^ with 45 kV and 40 mA of generator voltage and current. Operando XRD was performed during calcination in aerobic atmosphere using the Empyrean diffractometer equipped with a high-temperature furnace (Anton Paar HTK 1200 N, Anton Paar, Austria). Consequently, the XRD data were obtained by increasing or decreasing the temperature (10 °C min^–1^) with Cu–K*α*1 radiation in the 2*θ* range of 10–90° (2.5° min^–1^) after maintaining the temperature for 10 min at each step. Moreover, neutron diffraction (ND) pattern was obtained from the HANARO facility in the Korea Atomic Energy Research Institute (KAERI). The measurement was conducted in the 2θ range of 0–160° with a step size of 0.05 using a constant wavelength of 1.834578 Å. Additionally, the Raman spectra were obtained using an inVia Raman microscope (Renishaw, UK) equipped with a 514-nm-excitation-laser source (~1 mW power). Furthermore, XPS measurements were performed using a Quantum2000 spectrometer (ULVAC-PHI, Japan) with a monochromatized Al–K*α* source (1486.6 eV) operated at a power of 25 W at 15 kV. All the samples for the XPS measurements were sealed in dry room and transferred to an Ar-filled glove box, and then transferred to a vacuum chamber to avoid air exposure. The diameter of the X-ray spot was 100 μm. All measurements were performed in the fixed analyzer transmission mode, and the base pressure was under 1 × 10^–9^ Torr during the measurements. The survey scan spectra were recorded in the binding energy range from −2 to 1250 eV with an energy step width of 0.8 eV, and the detailed spectra were recorded for C 1s, La 3*d*, Zr 3*d*, Hf 4*f*, Sn 3*d*, and Sc 2*p* at a pass energy of 20.00 eV with an energy step width of 0.125 eV. C-K edge and O-K edge sXAS spectra in total electron yield (TEY) mode and fluorescence yield (FY) mode were recorded at the bending magnet beamline (10D XAS KIST) of Pohang Light Source-II (PLS-II) at Pohang Accelerator Laboratory (PAL). The sXAS spectra were collected using an X-ray beam of 3 mm × 1 mm size at ~25 °C under a base pressure of 1.5 × 10^−8^ Torr with 0.1 eV energy resolution. All the samples were sealed in aluminum-coated pouches in an Ar-filled glove box for the transfer to the beamline and mounted to the sample holder using a load-lock chamber to avoid air exposure. For the sample preparation, the solid electrolyte powder was sandwiched between two pieces of lithium metal foil (Honjo Metal Co., Ltd.) with 8 mm diameter and pressed under 2 tons for 10 sec in an Ar-filled glove box. The samples were stored in a vacuum chamber during 3 days. A Li foil piece was removed from one side of the laminated samples, and the samples were mounted on a sample holder.

### Computational details

All DFT calculations were conducted with Vienna Ab initio Simulation Package (VASP)^45^. In addition, generalized gradient approximation by Perdew–Burke–Ernzerhof^46^ was employed as an exchange-correlation functional, and the projector augmented wave pseudopotentials were used as distributed in VASP^47^. The cutoff energy for the plane wave basis set was set to 520 eV, and all other input parameters were generated with pymatgen^48^ to ensure that the results are compatible with the Materials Project (MP) database^49^. Thus, we could generate the Li grand potential phase diagram using MP entries and efficiently ensure the electrochemical stability of various garnets, as introduced in prior research^50,51^. Moreover, the structure relaxations were performed using the geometric information obtained by the Rietveld refinement of neutron diffraction to calculate enthalpy of each phase. As all configurations of the Li/vacancy and doped metal orderings cannot be practically calculated, we initially screened a handful of configurations using the order of Ewald summation energy, and thereafter, performed DFT calculations for the selected structures to identify the lowest energy structure. To calculate the cubic phases, we set the lattice parameter of the unit cell to prevent the structure from transiting into tetragonal symmetry. Subsequently, the optimal lattice parameter was determined by adjusting the cell size while maintaining the cell shape. Vibrational entropy was calculated using phonopy package^52^. To represent the random ordering of dopants, special quasirandom structures^53^ were generated using ATAT code^54^ for vibrational entropy calculation.

### Preparation and assembly of quasi-all-solid-state batteries

For the electrochemical measurement, sintered pellets were polished in the dry room (dew point, –60 °C) with 400-grit SiC abrasive paper. The thickness of pellets was 600–800 μm. After polishing, the pellet surface was washed with anhydrous ethanol and immediately dried with blowing of dry air. To measure the ionic conductivity, gold was sputtered on the garnet surface at 30 mA for 5 min for each surface (Cressington 208HR, Ted Pella Inc., USA). In addition, 20-μm-thick lithium metal foil (>99%, Honjo Metal Co., Ltd.) was attached to the garnet SEs in a dry-room and then cold isostatic pressure (CIP) of 250 MPa was applied for 3 min in a vacuum environment. The NCM111 cathode (loading density: 18.75 mg/cm^2^, active material weight fraction: 96 wt%; Samsung SDI) wetted with an ionic liquid electrolyte (2 M LiFSI in Pyr_13_FSI) of 3 μl was applied to the opposite side of the garnet solid electrolyte for the Li metal cell assembly. We used a cathode with 4 mm diameter corresponding to a capacity of ~0.402 mAh for the Li metal cell measurement. Thereafter, the cell was assembled in 2032-type coin cell (Hohsen Corp.) using manual coin cell hand crimper (Hohsen Corp.) by adding a spacer and spring in consideration of the thickness of solid electrolyte pellet. Moreover, an Li|SE|Li symmetric cell was assembled in 2032-type coin cell after applying the CIP to investigate the reduction stability of solid electrolytes. All the cell measurements were conducted without additional external pressure.

### Electrochemical measurement

Furthermore, potentiostatic electrochemical impedance spectroscopy (PEIS) measurements were performed to measure the ionic conductivity of solid electrolytes using a 1470E potentiostat/galvanostat and a 1455 frequency response analyzer (FRA) multichannel test module (Solatron Analytical, UK). All PEIS measurements were conducted at a constant potential mode with 10 mV AC amplitude and data was collected with ten frequencies per decade. A cell was kept under open-circuit potential for 10 min before carrying out the PEIS measurements. In particular, the PEIS was performed in the frequency range from 1 MHz to 0.1 Hz at various temperatures (25–60 °C) with the Au|SE|Au cell configuration. The ionic conductivities (*σ*_*Li*+_) were calculated from Nyquist plots Supplementary Fig. 4) using the following equation:

$${{{{{{\rm{\sigma }}}}}}}_{{{Li}}^{+}}=\frac{l}{{RA}}$$

where *R*, *A,* and *l*, correspond to the resistance, area of Au electrode, and thickness of SE pellets, respectively. The temporal variations in the PEIS of Li|SE|Li symmetric was evaluated every 32 min in the frequency range from 1 MHz to 0.01 Hz at 60 °C. Moreover, the galvanostatic charge and discharge profiles were measured at 60 °C with current densities of 0.2 and 0.8 mA/cm^2^ for the Li|SE|Li symmetric cell and Li|SE–IL|NCM111 cell during charge and discharge, respectively (TOSCAT-3100, Toyo System Co., Ltd., Fukushima, Japan). The current density of 0.1 mA/cm^2^ corresponds to 5.33 mA/g by considering only for the mass of active cathode material and the specific capacity is also based on the mass of cathode active material. The cycle-life of the acid-treated garnet was measured at a current density of 3.0 mA/cm^2^ at 60 °C, and the rate performance was measured with a current density from 0.6–2.2 mA/cm^2^ to 0.4–3.0 mA/cm^2^ for before and after acid treatment with an interval of 0.2 mA/cm^2^ at 60 °C, respectively. All the electrochemical test was performed several times at least over three times within the environmental chamber of constant temperature for reproducibility.

### Reporting summary

Further information on research design is available in the Nature Portfolio Reporting Summary linked to this article.

## Supplementary information

Supplementary Information

Reporting Summary

## Data Availability

The data generated or analyzed in this study are available from the corresponding authors upon reasonable request.
