# Supplementary material for: Unlocking the hidden chemical space in cubic-phase garnet solid electrolyte for efficient quasi-all-solid-state lithium batteries
Source: Nat Commun. 2022 Dec 10;13:7638. doi: 10.1038/s41467-022-35287-1 (PMC9741625; doi:10.1038/s41467-022-35287-1)
Supplement: Supplementary file 1 — Supplementary Information [file 41467_2022_35287_MOESM1_ESM.pdf]

Supplementary Information

Unlocking the hidden chemical space in cubic-phase garnet solid electrolyte for efficient quasi-all-solid-state lithium batteries

Sung-Kyun Jung[1,2,†,*], Hyeokjo Gwon[1,†,*], Hyungsub Kim[3], Gabin Yoon[1], Dongki Shin[4], Jihyun Hong[4], Changhoon Jung[5], and Ju-Sik Kim[1]

Affiliation

[1] Battery Material Lab, Material Research Center, Samsung Advanced Institute of Technology (SAIT), Samsung Electronics Co., Ltd., 130 Samsung-ro, Yeongtong-gu, Suwon-si, Gyeonggi-do, 16678 Republic of Korea

[2] School of Energy and Chemical Engineering, Ulsan National Institute of Science and Technology (UNIST), 50 UNIST-gil, Ulsan, 44919 Republic of Korea

[3] Neutron Science Center, Korea Atomic Energy Research Institute (KAERI), 111 Daedeok-daero 989 Beon-Gil, Yuseong-gu, Daejeon, 34057 Republic of Korea

[4] Energy Materials Research Center, Korea Institute of Science and Technology (KIST), Seoul, 02792 Republic of Korea

[5] Analytical Engineering Group, Material Research Center, Samsung Advanced Institute of Technology (SAIT), Samsung Electronics Co., Ltd., 130 Samsung-ro, Yeongtong-gu, Suwon-si, Gyeonggi-do, 16678 Republic of Korea

* Corresponding to: skjung@unist.ac.kr, h.gwon@samsung.com

†These authors contributed equally to this work.

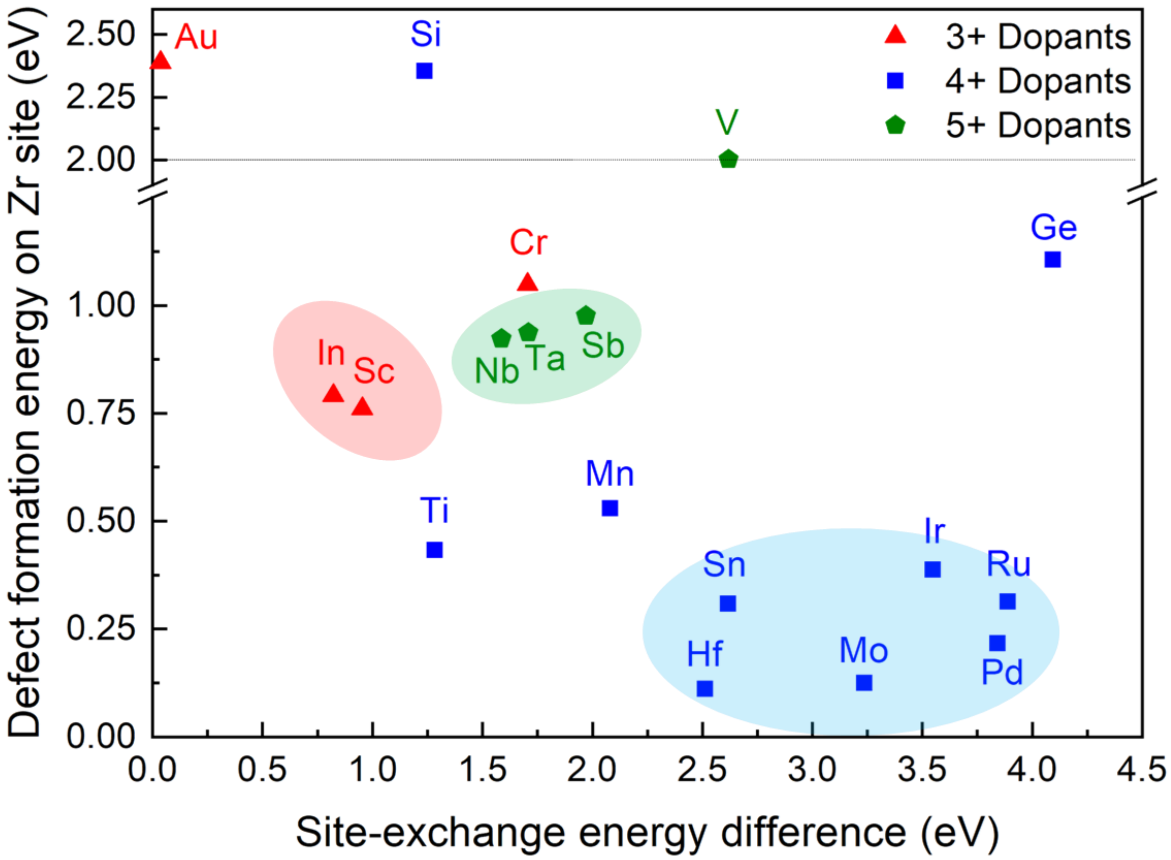

Supplementary Figure 1. Dopant species with preference on Zr site in $Li_7La_3Zr_2O_{12}$ garnet phase. Site-exchange energy difference represents the minimum difference in defect formation energy between Zr and Li or La sites. Dopants with low defect formation energy and large difference in site-exchange energy can conveniently substitute Zr site. Shaded regions represent the most preferred dopants on Zr site with various oxidation states. (Reproduced with permission from L. J. Miara *et al.*, *Chem. Mater.* **27** (2015) 4040[1]. Copyright © 2015 American Chemical Society)

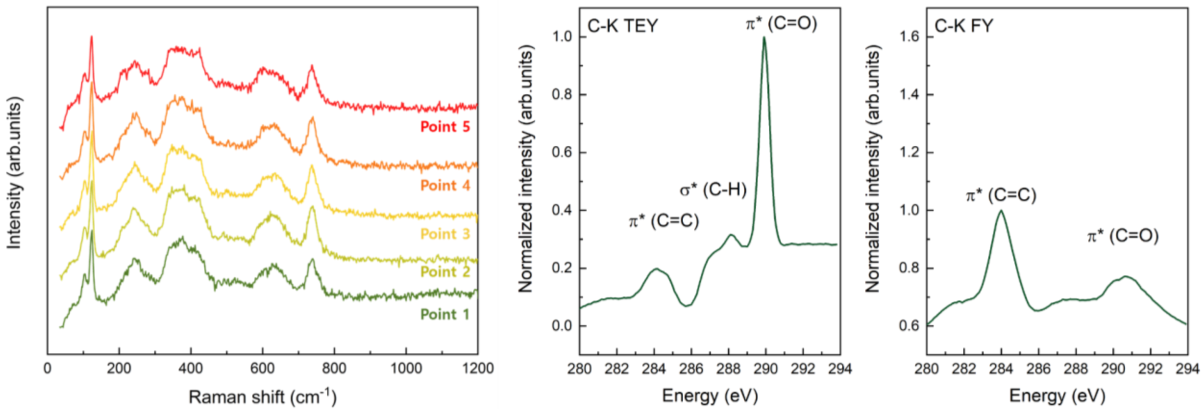

Supplementary Figure 2. (Left) Raman spectra of $Li_7La_3Zr_{0.4}Hf_{0.4}Sn_{0.4}Sc_{0.4}Ta_{0.4}O_{12}$ at multiple points and (Right) soft X-ray absorption spectroscopy spectra at C-K edge collected in total electron yield (TEY) and fluorescence yield (FY) modes.

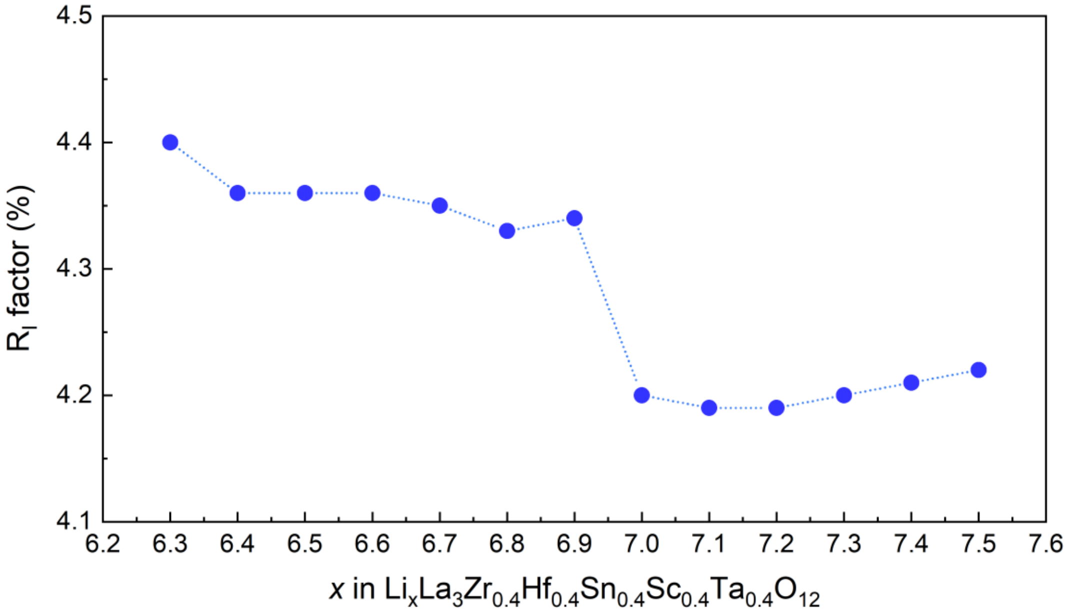

Supplementary Figure 3. R_I factors according to lithium contents obtained from neutron diffraction Rietveld refinement result of the Li = 7.0 garnet SE.

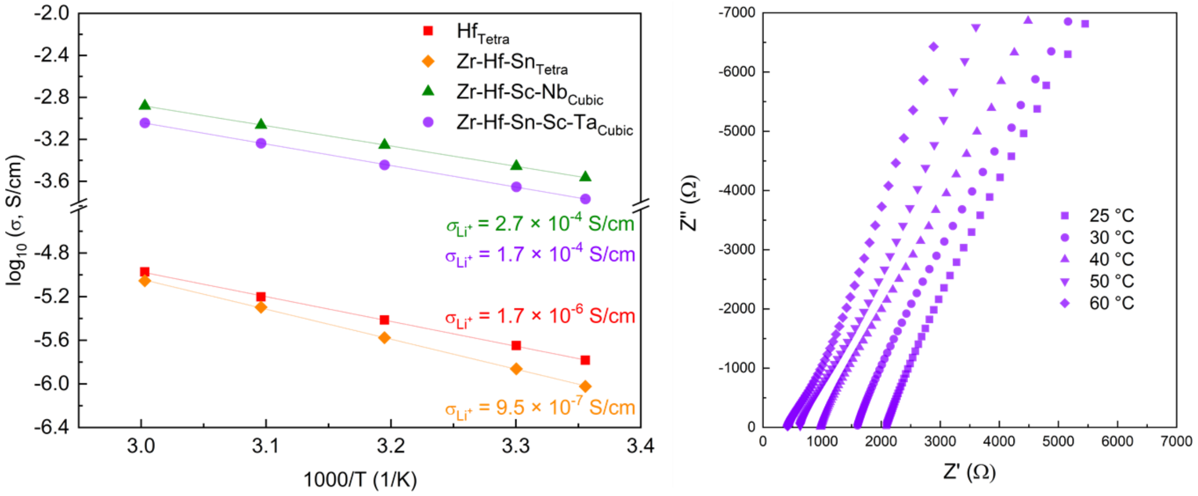

Supplementary Figure 4. (Left) Arrhenius plot of tetragonal and cubic garnet phases, and their lithium ionic conductivities at room temperature. (Right) Nyquist plots of the Li = 7.0 garnet SEs at the temperature range of 25–60 °C.

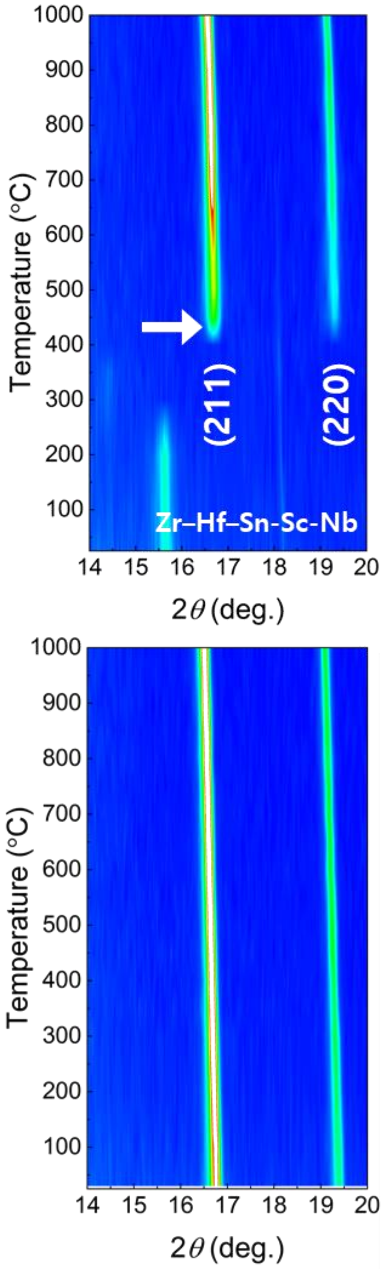

Supplementary Figure 5. Operando phase evolution during calcination of $Li_7La_3Zr_{0.4}Hf_{0.4}Sn_{0.4}Sc_{0.4}Nb_{0.4}O_{12}$; top: contour plot of X-ray diffraction patterns during heating process; bottom: cooling process in range of 14–20°.

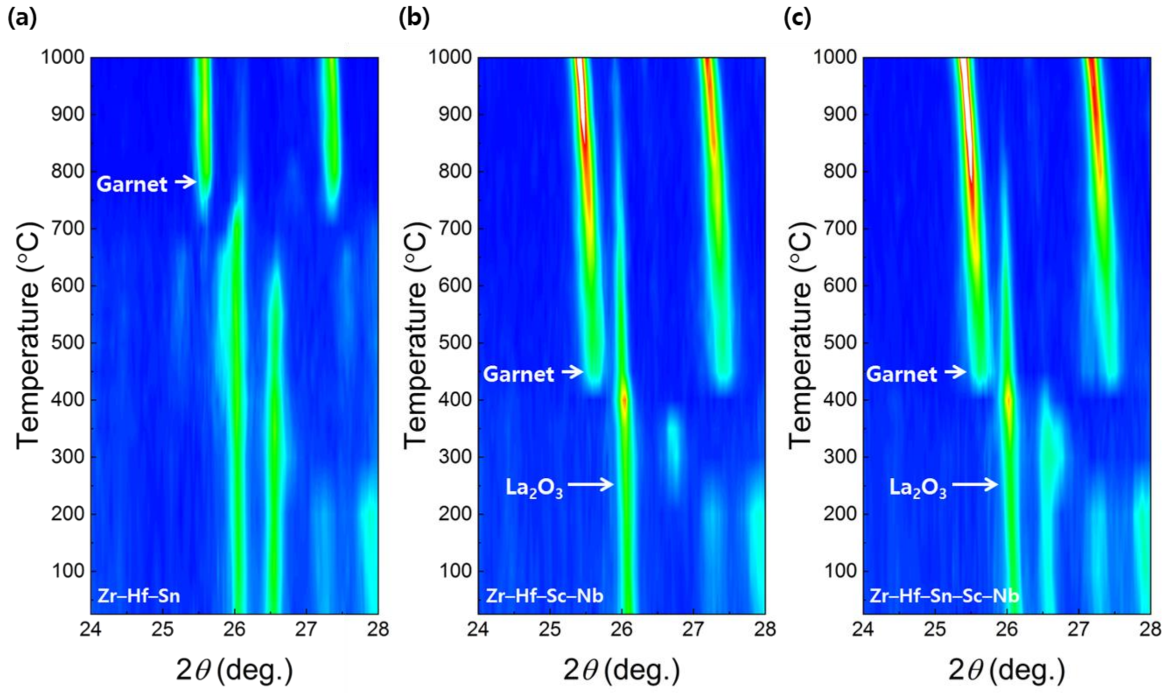

Supplementary Figure 6. Operando phase evolution and contour plot of X-ray diffraction during heating process of (a) $Li_7La_3Zr_{2/3}Hf_{2/3}Sn_{2/3}O_{12}$, (b) $Li_7La_3Zr_{0.5}Hf_{0.5}Sc_{0.5}Nb_{0.5}O_{12}$ and (c) $Li_7La_3Zr_{0.4}Hf_{0.4}Sn_{0.4}Sc_{0.4}Nb_{0.4}O_{12}$ in range of 24–28°.

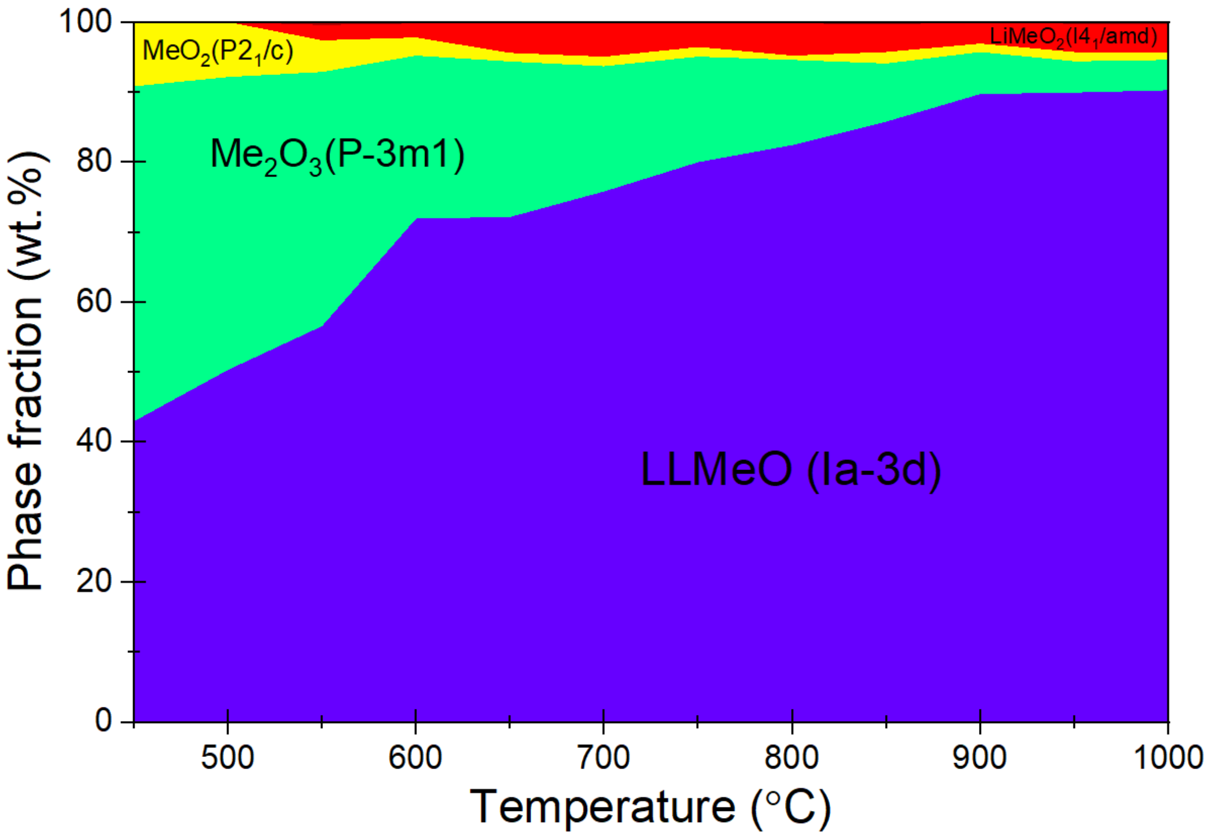

Supplementary Figure 7. Phase fractions present in $Li_7La_3Zr_{0.4}Hf_{0.4}Sn_{0.4}Sc_{0.4}Ta_{0.4}O_{12}$ during heating process.

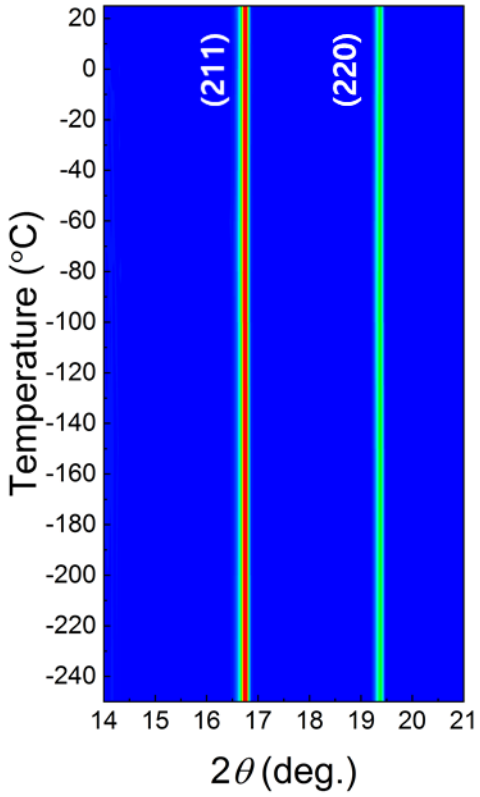

Supplementary Figure 8. Operando X-ray diffraction of $Li_7La_3Zr_{0.4}Hf_{0.4}Sn_{0.4}Sc_{0.4}Ta_{0.4}O_{12}$ during cooling process from 20 °C to –253 °C.

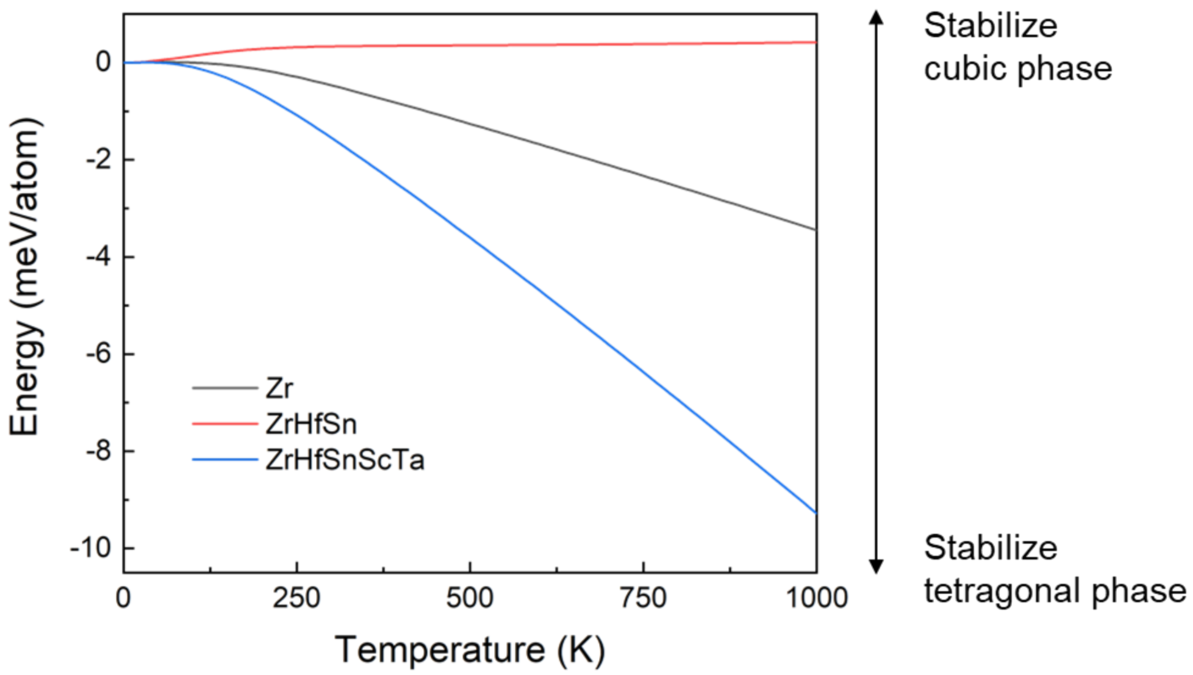

Supplementary Figure 9. Difference in energy contribution from vibrational entropy obtained by DFT calculation ($T \times S_{vib,cubic} - T \times S_{vib,tetra}$).

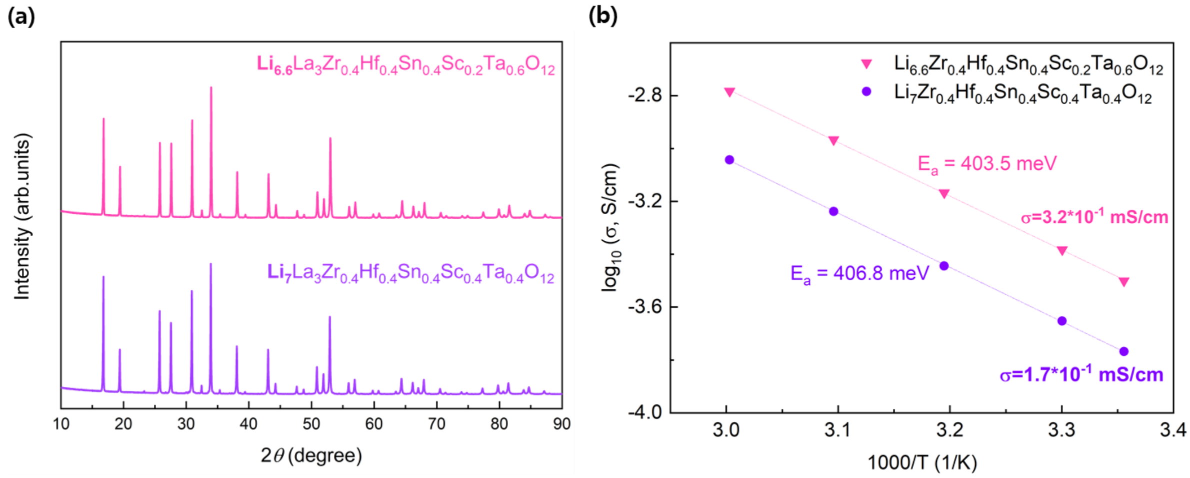

Supplementary Figure 10. (a) X-ray diffraction patterns of $Li_{6.6}La_3Zr_{0.4}Hf_{0.4}Sn_{0.4}Sc_{0.2}Ta_{0.6}O_{12}$; (b) Arrhenius plot of ionic conductivity as a function of temperature.

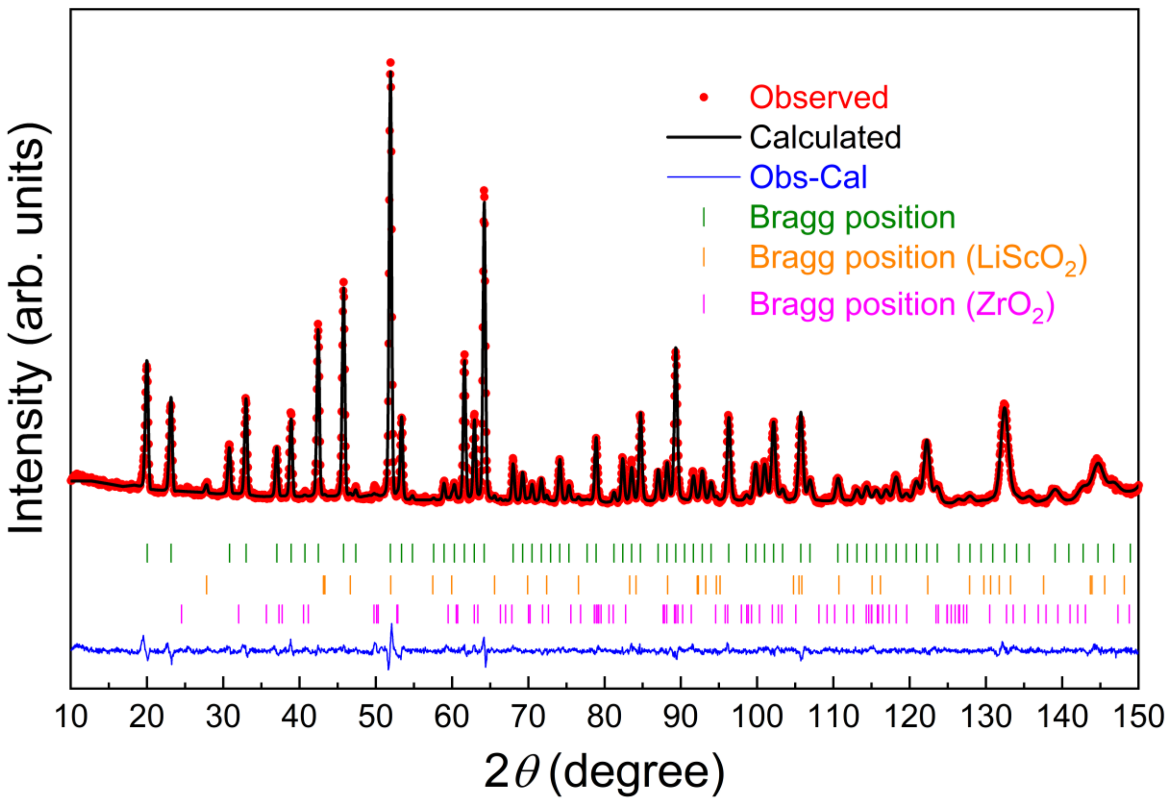

Supplementary Figure 11. Neutron diffraction pattern and Rietveld refinement of $Li_{6.6}La_3Zr_{0.4}Hf_{0.4}Sn_{0.4}Sc_{0.2}Ta_{0.6}O_{12}$.

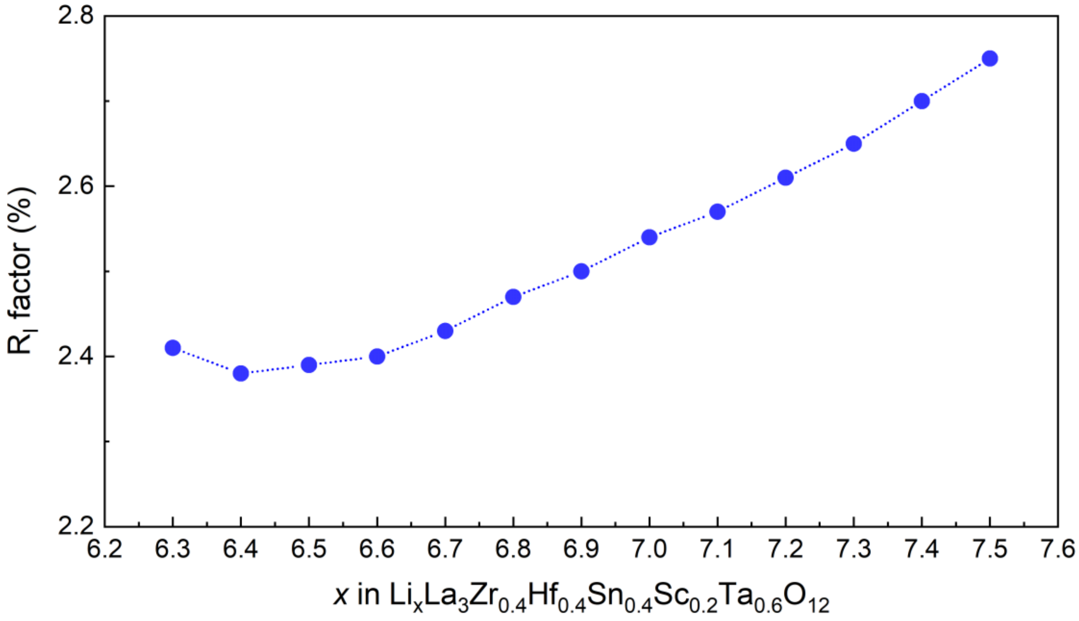

Supplementary Figure 12. R_I factors according to lithium contents obtained from neutron diffraction Rietveld refinement result of Li = 6.6 garnet.

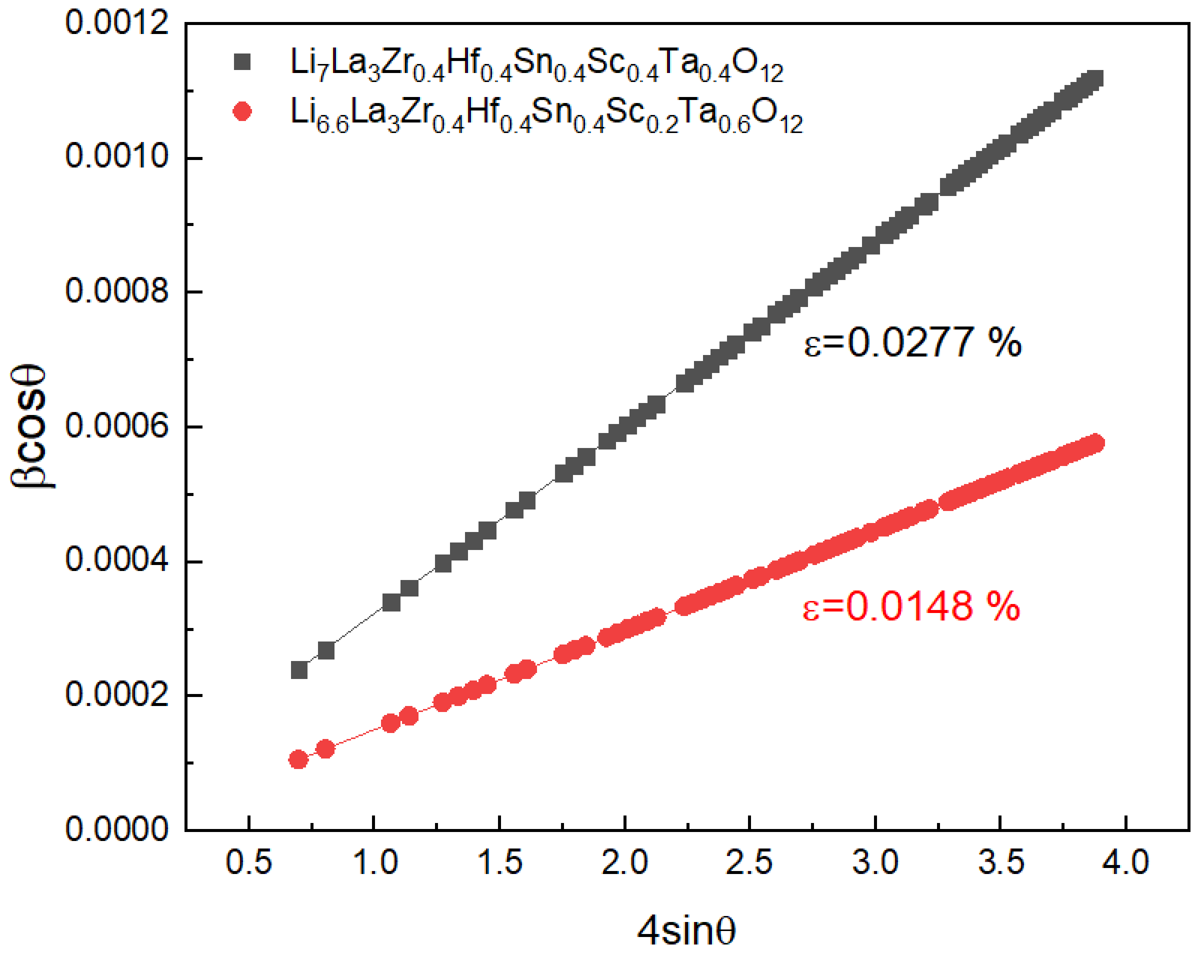

Supplementary Figure 13. Williamson–Hall plots of $Li_7La_3Zr_{0.4}Hf_{0.4}Sn_{0.4}Sc_{0.4}Ta_{0.4}O_{12}$ and $Li_{6.6}La_3Zr_{0.4}Hf_{0.4}Sn_{0.4}Sc_{0.2}Ta_{0.6}O_{12}$ from neutron diffraction Rietveld refinement.

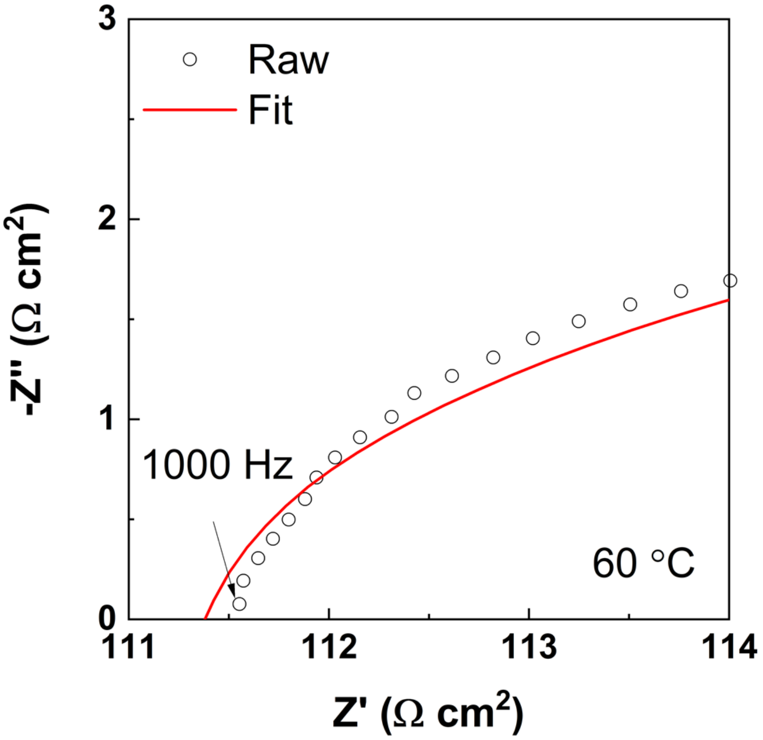

Supplementary Figure 14. Enlarged EIS spectra of Li||Li symmetric cell for the Li=7.0 garnet (60 ℃) at high frequency region without additional external pressure.

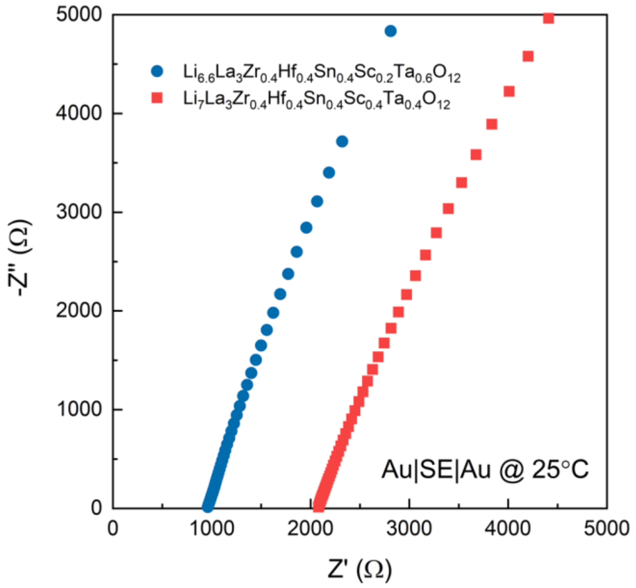

Supplementary Figure 15. EIS measurements of $Li_7La_3Zr_{0.4}Hf_{0.4}Sn_{0.4}Sc_{0.4}Ta_{0.4}O_{12}$ and $Li_{6.6}La_3Zr_{0.4}Hf_{0.4}Sn_{0.4}Sc_{0.2}Ta_{0.6}O_{12}$ in Au|SE|Au configuration at 25 ℃ without external pressure.

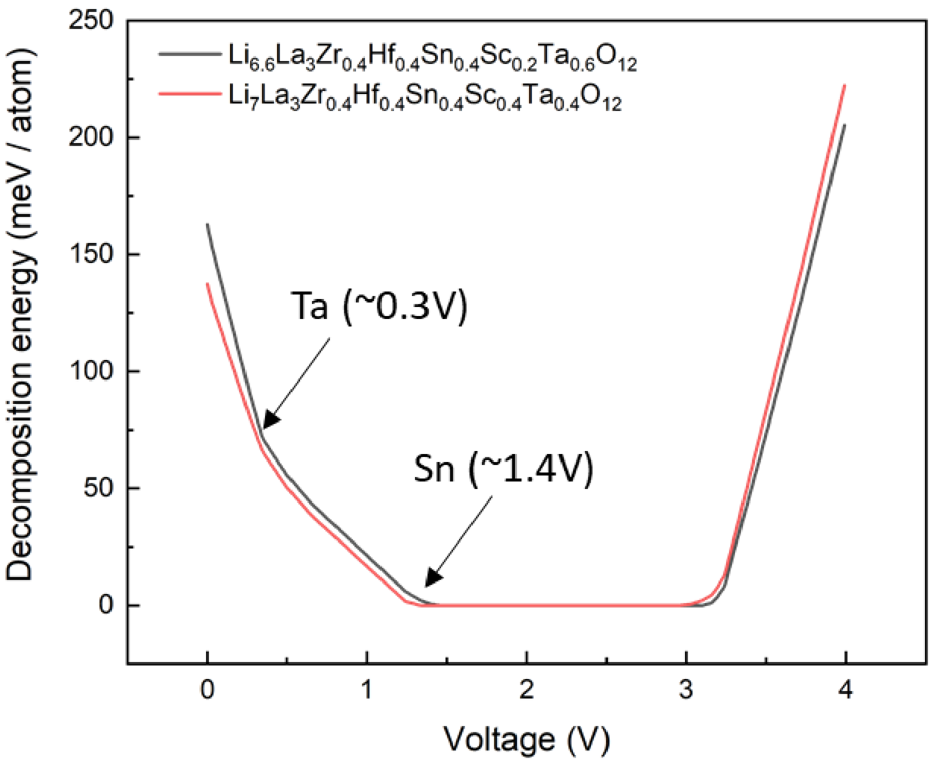

Supplementary Figure 16. Thermodynamic stability window of the Li = 7.0 and Li = 6.6 garnet SEs containing multidopants in Zr site.

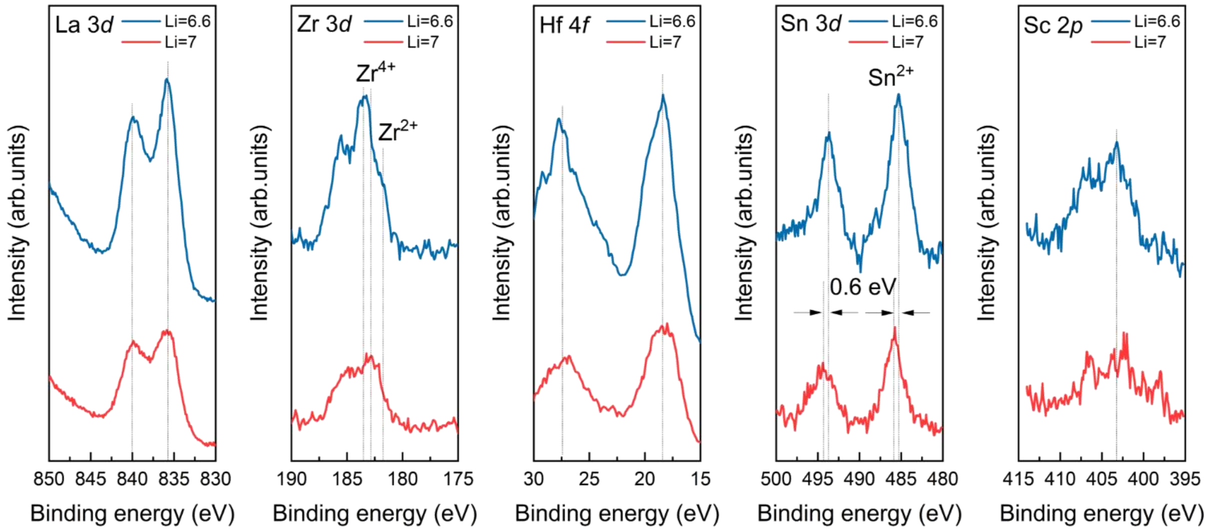

Supplementary Figure 17. XPS spectra of the Li = 7.0 and Li = 6.6 garnet SEs after contacting with lithium metal. The lithium metal was attached to the SEs in a dry-room (dew point, –60 °C) and then cold isostatic pressure (CIP) of 250 MPa was applied for 3 min in a vacuum environment.

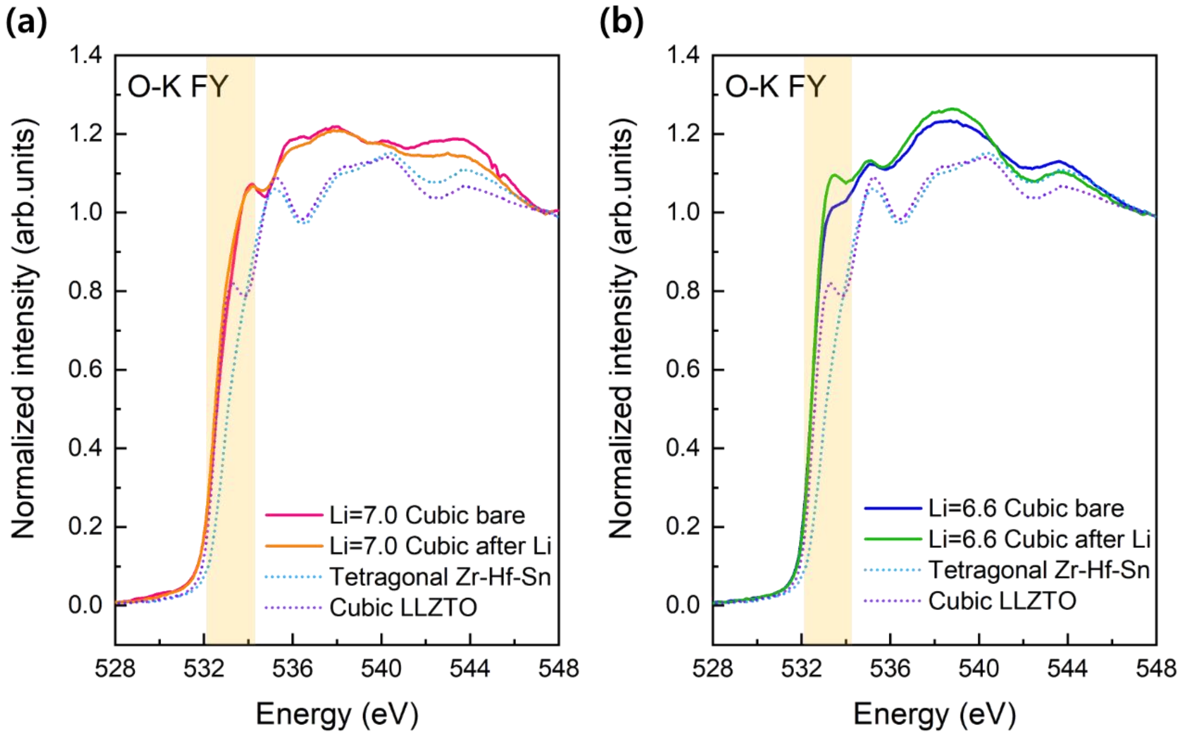

Supplementary Figure 18. O-K edge soft X-ray absorption spectroscopy spectra of (a) the Li = 7.0 and (b) Li = 6.6 garnet SEs collected in fluorescence yield (FY) mode before and after contact with Li metal (see Method in the manuscript for the detail sample preparation process). Dotted lines indicate the reference spectra of tetragonal-phase garnet ($Li_7La_3Zr_{2/3}Hf_{2/3}Sn_{2/3}O_{12}$) and Ta-doped cubic-phase garnet ($Li_{6.5}La_3Zr_{1.5}Ta_{0.5}O_{12}$).

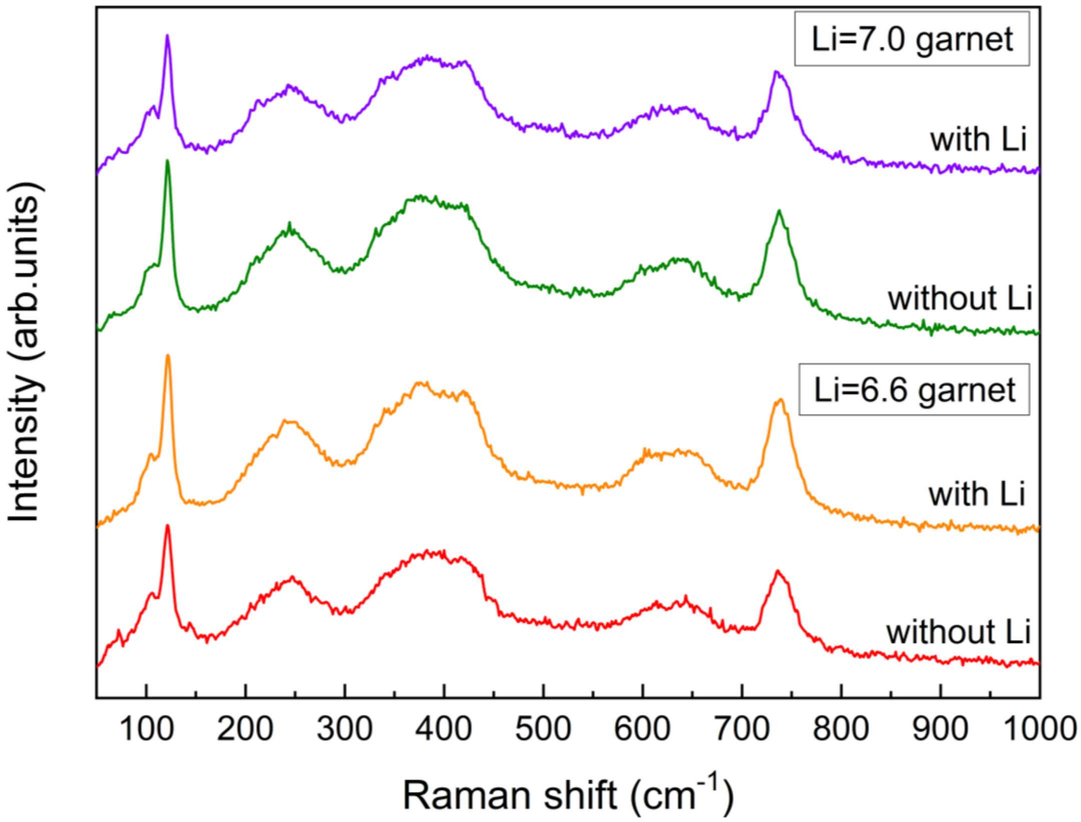

Supplementary Figure 19. Raman spectra of the Li = 7.0 and Li = 6.6 garnet SEs before and after contacting with lithium metal. The lithium metal was attached to the SEs in a dry-room (dew point, –60 °C) and then cold isostatic pressure (CIP) of 250 MPa was applied for 3 min in a vacuum environment.

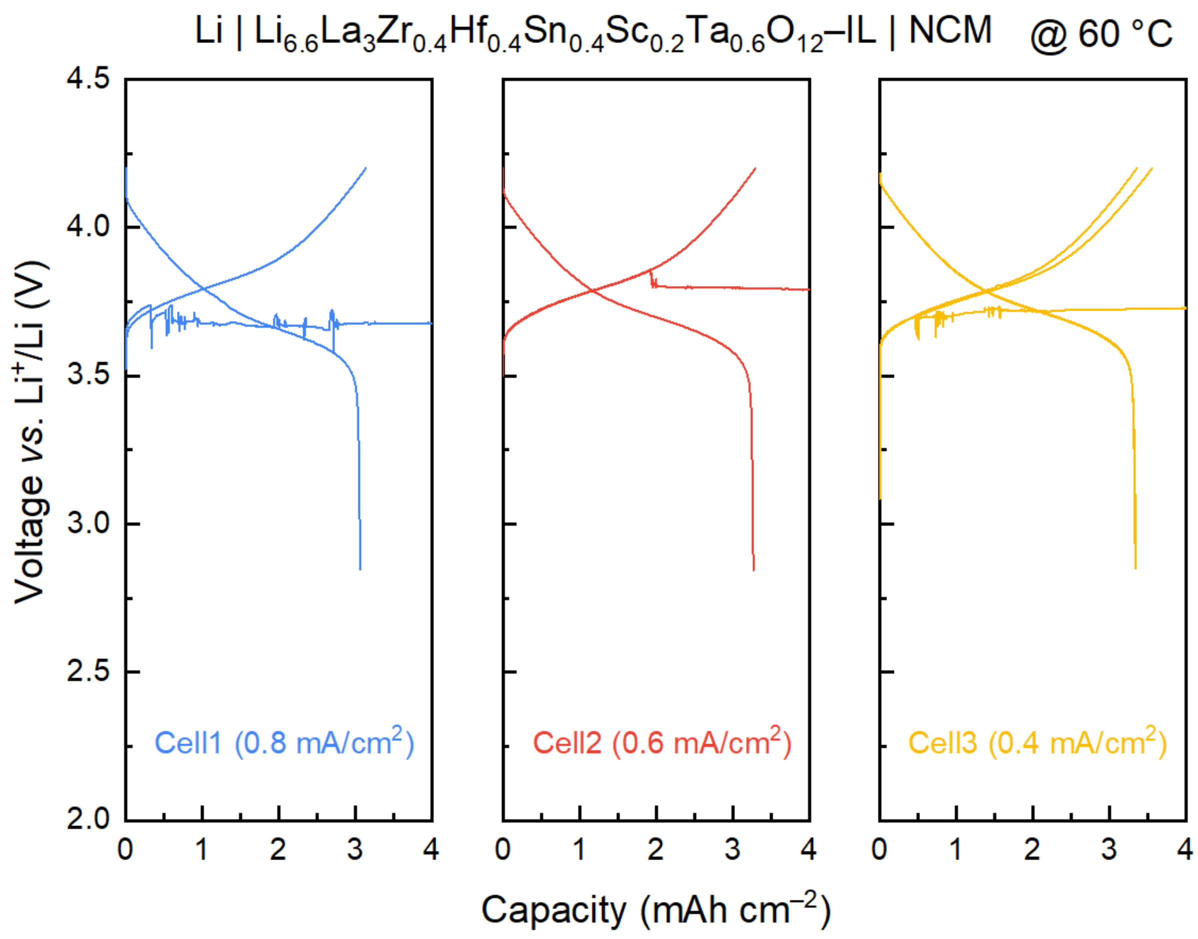

Supplementary Figure 20. Charge–discharge profiles of the coin cells comprising the Li = 6.6 garnet SE at various current densities without additional external pressure, and their short-circuit at early stages of cycle.

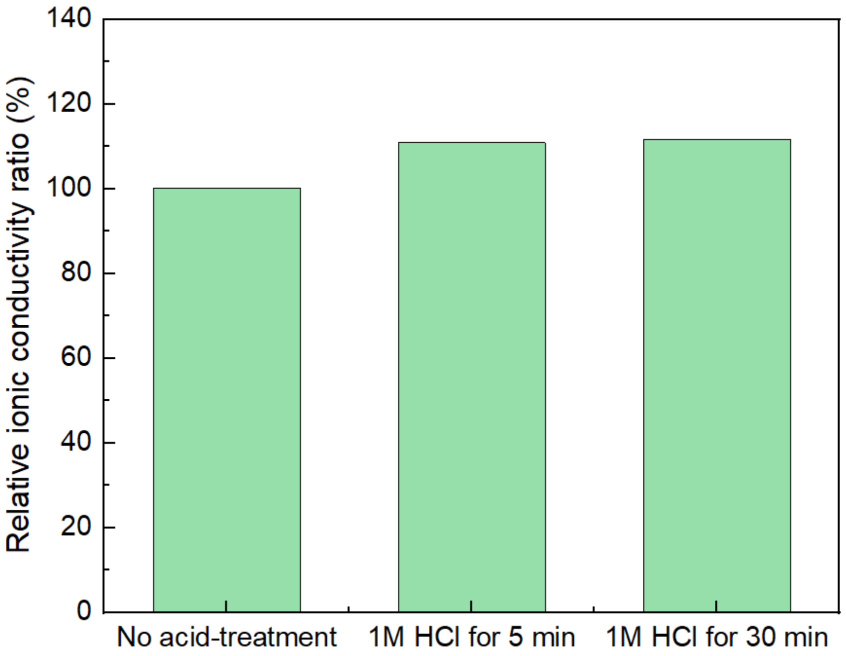

Supplementary Figure 21. Relative ionic conductivity of the Li = 7.0 garnet SEs before and after acid-treatment

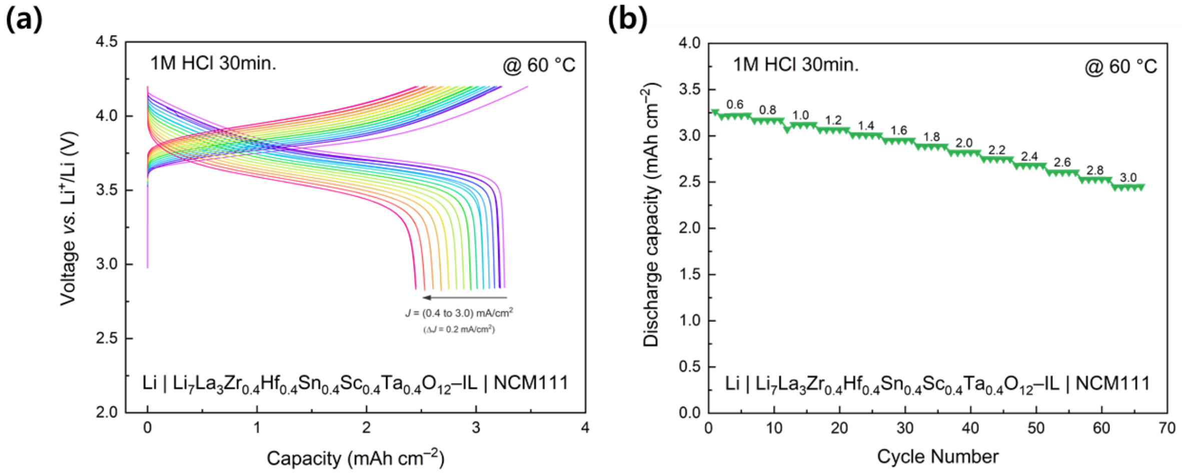

Supplementary Figure 22. Rate performance of Li||NCM111 coin cell comprising acid-treated (1M HCl for 30 min) entropy-driven Li = 7.0 cubic-phase garnet SE. (a) Electrochemical profiles at various current densities (0.4–3.0 mA/cm^2 with an interval of 0.2 mA/cm^2), and (b) its capacity retention during 5 cycles at various current densities. The rate performance was measured without additional external pressure.

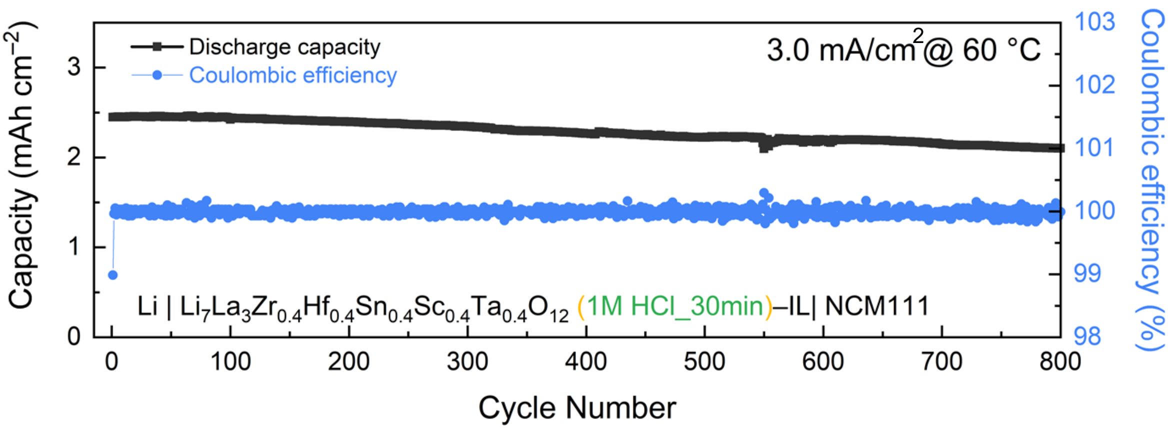

Supplementary Figure 23. Long-term cycling performance of Li||NCM111 coin cell comprising acid-treated (1M HCl for 30 min) entropy-driven Li = 7.0 cubic-phase garnet SE at high current density (*J*) of 3.0 mA/cm^2 after rate test in Supplementary Fig. 22. The cell was cycled without additional external pressure.

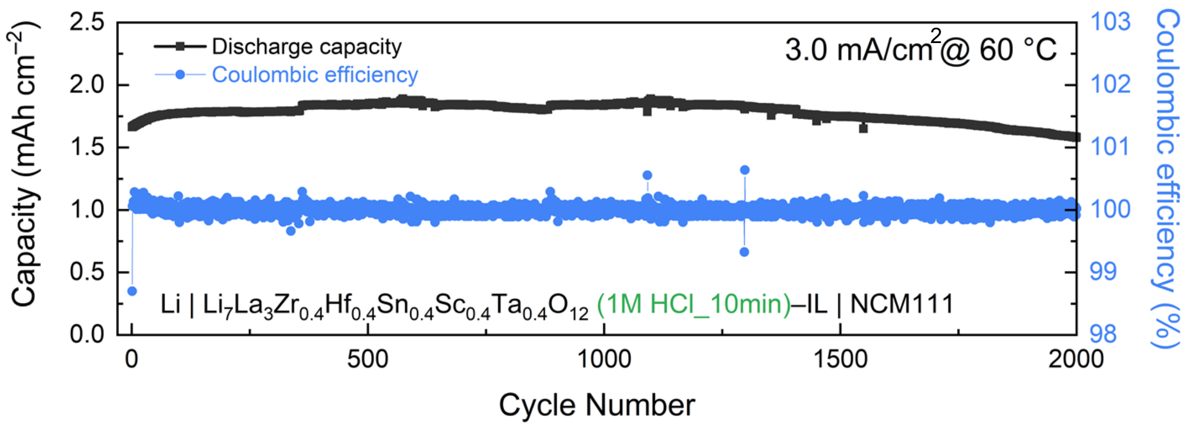

Supplementary Figure 24. Long-term cycling performance of Li||NCM111 coin cell comprising acid-treated (1M HCl for 10 min) entropy-driven Li = 7.0 cubic-phase garnet SE at high current density (*J*) of 3.0 mA/cm^2. The cell was cycled without additional external pressure.

Supplementary Table 1. Composition of atomic ratio in garnets obtained by ICP-AES measurements.

Samples	ICP-AES (atomic fraction)							
	**Li**	**La**	**Zr**	**Hf**	**Sn**	**Sc**	**Ta**	**Nb**
$\mathbf{T_{Zr–Hf–Sn}}$	7.173	3	0.581	0.677	0.653	–	–	–
$\mathbf{C_{Zr–Hf–Sc–Nb}}$	7.168	3	0.566	0.492	–	0.504	–	0.516
$\mathbf{C_{Zr–Hf–Sn–Sc–Ta}}$	7.247	3	0.420	0.407	0.407	0.407	0.420	–
$\mathbf{C_{Zr–Hf–Sn–Sc–Nb}}$	7.609	3	0.396	0.421	0.409	0.409	–	0.447

Supplementary Table 2. X-ray diffraction Rietveld refinement results of $Li_7La_3Zr_{0.4}Hf_{0.4}Sn_{0.4}Sc_{0.4}Ta_{0.4}O_{12}$ based on Li-site occupancy with Neutron diffraction Rietveld refinement result.

Site	Multiplicity	x	y	z	B_{iso}	Occupancy
Li1	24	0.375	0	0.25	0.83228	0.468
Li2	96	0.69161	0.59754	0.11431	5.08863	0.466
La1	24	0.125	0	0.25	1.101(17)	1
Zr1	16	0	0	0	0.626(19)	0.2
Hf1	16	0	0	0	0.626(19)	0.2
Sn1	16	0	0	0	0.626(19)	0.2
Sc1	16	0	0	0	0.626(19)	0.2
Ta1	16	0	0	0	0.626(19)	0.2
O1	96	0.2823(2)	0.1027(2)	0.1964(3)	1.46(10)	1

Supplementary Table 3. Phase fraction in $Li_7La_3Zr_{0.4}Hf_{0.4}Sn_{0.4}Sc_{0.4}Ta_{0.4}O_{12}$ based on Neutron diffraction Rietveld refinement results.

Sample	Phase	Phase fraction (wt.%)	R_I(%)	R_F(%)
$Li_7La_3Zr_{0.4}Hf_{0.4}Sn_{0.4}Sc_{0.4}Ta_{0.4}O_{12}$<br>$R_p$: 3.58 %<br>$R_{wp}$: 4.67 %	Cubic	97.89 (1.19)	4.20	3.26
	$LiScO_2$	0.99 (0.04)	–	–
	ZrO_2	1.12 (0.15)	–	–

Supplementary Table 4. Neutron diffraction Rietveld refinement results of $Li_7La_3Zr_{0.4}Hf_{0.4}Sn_{0.4}Sc_{0.4}Ta_{0.4}O_{12}$.

Site	Multiplicity	x	y	z	B_{iso}	Occupancy
Li1	24	0.375	0	0.25	0.5(6)	0.41(2)
Li2	96	0.690(1)	0.579(1)	0.097(1)	1.2(3)	0.48(1)
La1	24	0.125	0	0.25	0.56(5)	1
Zr1	16	0	0	0	1.35(7)	0.2
Hf1	16	0	0	0	1.35(7)	0.2
Sn1	16	0	0	0	1.35(7)	0.2
Sc1	16	0	0	0	1.35(7)	0.2
Ta1	16	0	0	0	1.35(7)	0.2
O1	96	0.2815(1)	0.1007(2)	0.1962(2)	1.07(5)	1

Supplementary Table 5. Formation energy differences between cubic and tetragonal phases.

**Energy (meV/atom)**	**Zr**	**Zr–Hf–Sn**	**Zr–Hf–Sc–Nb**	**Zr–Hf–Sn–Sc–Ta**
$\mathbf{H_{Cubic}–H_{Tetra}}$	3.70	4.38	5.48	4.54

Supplementary Table 6. Phase fraction in $Li_{6.6}La_3Zr_{0.4}Hf_{0.4}Sn_{0.4}Sc_{0.2}Ta_{0.6}O_{12}$ based on neutron diffraction Rietveld refinement results.

Sample	Phase	Phase fraction (wt.%)	R_I(%)	R_F(%)
$Li_{6.6}La_3Zr_{0.4}Hf_{0.4}Sn_{0.4}Sc_{0.2}Ta_{0.6}O_{12}$<br>$R_p$: 3.03 %<br>$R_{wp}$: 3.89 %	Cubic	98.96 (0.69)	2.4	2.02
	$LiScO_2$	0.38 (0.02)	–	–
	ZrO_2	0.66 (0.11)	–	–

Supplementary Table 7. Neutron diffraction Rietveld refinement results of $Li_{6.6}La_3Zr_{0.4}Hf_{0.4}Sn_{0.4}Sc_{0.2}Ta_{0.6}O_{12}$.

Site	Multiplicity	x	y	z	B_{iso}	Occupancy
Li1	24	0.375	0	0.25	3.8(5)	0.51(3)
Li2	96	0.6875(9)	0.5805(8)	0.0963(8)	2.5(3)	0.42(1)
La1	24	0.125	0	0.25	0.70(3)	1
Zr1	16	0	0	0	0.81(4)	0.2
Hf1	16	0	0	0	0.81(4)	0.2
Sn1	16	0	0	0	0.81(4)	0.2
Sc1	16	0	0	0	0.81(4)	0.1
Ta1	16	0	0	0	0.81(4)	0.3
O1	96	0.28130(8)	0.10165(8)	0.19725(9)	0.99(2)	1

Supplementary Table 8. Fitting parameters for the initial EIS spectrum (Figs. 4a and 4b in the manuscript) of Li|SE|Li symmetric cell.

**Li|Garnet|Li**	**Li=7.0 garnet** ($\chi^2 = 4.11 \times 10^{-5}$)		**Li=6.6 garnet** ($\chi^2 = 6.4 \times 10^{-5}$)	
	**Fit values**	**Error (%)**	**Fit value**	**Error (%)**
**L1 (H)**	2.1×10^{-4}	9.03	1.8×10^{-5}	2.43
**R1 (Ω)**	390.9	0.07	152.7	0.58
**R2 (Ω)**	-	-	56.1	1.87
Q_{CPE2} **(F s$^{\alpha-1}$)**	-	-	5.6×10^{-7}	6.57
α_{CPE2}	-	-	0.80	0.94
**R3 (Ω)**	48.47	1.93	71.46	1.2
Q_{CPE3} **(F s$^{\alpha-1}$)**	9.2×10^{-3}	1.84	6.5×10^{-3}	1.42
α_{CPE3}	0.37	2.01	0.44	1.14

Supplementary Note 1

Raman Spectroscopy

The Li=7.0 garnet exhibited typical Raman spectra of previously reported cubic-garnet phase except for the broad features at around 400 cm^{-1} Raman Shift[2]. The origin of Raman shift at 300–500 cm^{-1} was reported as induced by the vibration mode of Li in LiO_4 tetrahedron[3], therefore, the peak broadening in the region is presumably owing to the diversity of Li–O bonding environment in the LiO_4 tetrahedron caused by the multiple dopants.

Supplementary Note 2

Existence of Li_2CO_3 on garnet surface

Although the signal of Li_2CO_3 was not detected via Raman spectroscopy measurements, C=O bonding suspected from Li_2CO_3 was observed from XPS spectra. Given the sample preparation atmosphere (dry room), sensitivity of Raman and XPS instruments, a trace of amount Li_2CO_3 with a few nanometers thicknesses seems to exist. We further confirmed the existence of a few nanometers of Li_2CO_3 layer on the surface of the garnet using soft X-ray absorption spectroscopy (sXAS). The sXAS spectra acquired at C-K edge with total electron yield mode which is sensitive to a few nanometers (below 10 nm) showed π^* signal from C=O bonding. Meanwhile, the spectra with fluorescence yield mode which is sensitive to tens of nanometers (10–50 nm) thickness show dramatically decreased intensity of C=O bond. These results corroborate that trace amount of Li_2CO_3 corresponding to a few nanometer-thick exist on the surface.

Supplementary References

1 Miara, L. J., Richards, W. D., Wang, Y. E. & Ceder, G. First-principles studies on cation dopants and electrolyte| cathode interphases for lithium garnets. *Chem. Mater.* **27**, 4040-4047 (2015).

2 Larraz, G., Orera, A. & Sanjuan, M. Cubic phases of garnet-type $Li_7La_3Zr_2O_{12}$: the role of hydration. *J. Mater. Chem. A* **1**, 11419-11428 (2013).

3 Narayanan, S., Hitz, G. T., Wachsman, E. D. & Thangadurai, V. Effect of excess Li on the structural and electrical properties of garnet-type Li6La3Ta1. 5Y0. 5O12. *Journal of The Electrochemical Society* **162**, A1772 (2015).
